# Combined effects of hydrological conditions and socioeconomic factors on the seasonal dynamics of severe fever with thrombocytopenia syndrome in China, 2011–2022: a modelling study

**DOI:** 10.1016/j.lanwpc.2025.101564

**Published:** 2025-04-28

**Authors:** Hong-Han Ge, Kun Liu, Fang-Yu Ding, Peng Huang, Yan-Qun Sun, Ming Yue, Hong Su, Qian Wang, Nicholas Philip John Day, Richard James Maude, Dong Jiang, Li-Qun Fang, Wei Liu

**Affiliations:** a School of Public Health, Shandong First Medical University and Shandong Academy of Medical Sciences, Jinan, China; b State Key Laboratory of Pathogen and Biosecurity, Academy of Military Medical Science, Beijing, China; c Department of Epidemiology, The Ministry of Education Key Lab of Hazard Assessment and Control in Special Operational Environment, The Shaanxi Provincial Key Laboratory of Environmental Health Hazard Assessment and Protection, School of Public Health, The Fourth Military Medical University, Xi'an, China; d Institute of Geographic Sciences and Natural Resources Research, Chinese Academy of Sciences, Beijing, China; e College of Resources and Environment, University of Chinese Academy of Sciences, Beijing, China; f Department of Epidemiology, Center for Global Health, School of Public Health, National Vaccine Innovation Platform, Nanjing Medical University, Nanjing, China; g Clinical Research Center, Children's Hospital of Nanjing Medical University, Nanjing, China; h Department of Infectious Diseases, The First Affiliated Hospital of Nanjing Medical University, Nanjing, China; i School of Public Health, Anhui Medical University, Hefei, China; j Nuffield Department of Medicine, Centre for Tropical Medicine and Global Health, University of Oxford, Oxford, UK; k Mahidol Oxford Tropical Medicine Research Unit, Faculty of Tropical Medicine, Mahidol University, Bangkok, Thailand

**Keywords:** Severe fever with thrombocytopenia syndrome, Tick-borne disease, Hydrological conditions, Extreme climate, Socioeconomic factors, Exposure-lag-response association

## Abstract

**Background:**

Severe fever with thrombocytopenia syndrome (SFTS) is a tick-borne viral hemorrhagic fever with expanding geographical range. The determinants of the seasonal dynamics of SFTS remain poorly understood.

**Methods:**

Monthly SFTS cases from 604 counties in five provinces with high-notification rate in China (2011–2022) were analyzed using hierarchical Bayesian spatiotemporal and distributed lag nonlinear models. Cumulative and month-specific effects of meteorological factors were assessed, with socioeconomic factors as modifiers.

**Findings:**

The cumulative effect peaked at 21.97 °C (*RR* = 1.24, 95% CI: 1.10–1.40) and the month-specific effect peaked at 25.67 °C (*RR* = 1.38, 95% CI: 1.26–1.51) without time lag. Increased precipitation significantly amplified the risk of SFTS with a notable lag effect observed. Both drought and wet conditions heightened the risk of SFTS occurrence substantially, with cumulative *RR* peaking at 3.13 (95% CI: 1.58–6.23) for Standardized Precipitation Evapotranspiration Index (SPEI-1) of −2.5, indicating drought conditions, and peaking at 1.51 (95% CI: 1.00–2.27) for SPEI-1 of 2.16, indicating wet conditions. The highest month-specific *RR* was observed at an SPEI-1 of −2.5 with a 2-month lag and at 1.81 with a 1-month lag, respectively. The risk of SFTS was higher in low-urbanization areas during drought, while was higher in high-urbanization areas with wet conditions.

**Interpretation:**

Climatic factors significantly influence SFTS dynamics, with socioeconomic conditions modifying these effects. Integrating climate factors into surveillance and early warning systems is essential for targeted prevention and control.

**Funding:**

10.13039/501100001809National Natural Science Foundation of China (No. 82330103 and No. 42201497), 10.13039/501100012492Youth Innovation Promotion Association (No. 2023000117), and the 10.13039/100010269Wellcome Trust [220211].

**Research in context**

**Evidence before this study**

Meteorological factors, such as temperature and precipitation, have been recognized as key drivers of severe fever with thrombocytopenia syndrome (SFTS) incidence. We searched PubMed database on Jan 12th, 2025 by terms “(((SFTS) OR (severe fever with thrombocytopenia syndrome)) AND ((hydrological) OR (climate))) AND (socioeconomic)”, without language restrictions. Among the three articles retrieved, existing studies lacked a detailed examination of the combined effects of climatic and socioeconomic factors across diverse administrative regions in China. Additionally, the potential modifying influence of urbanization and socioeconomic conditions on these relationships has not been adequately explored.

**Added value of this study**

This study provides a novel, integrated assessment of the combined effects of hydrological conditions and socioeconomic factors on the seasonal patterns of SFTS over a 12-year period in China. Using hierarchical Bayesian spatiotemporal models and distributed lag nonlinear models, we identified nonlinear, lag-dependent relationships between SFTS and climatic variables, such as drought and wet conditions. Our findings highlight the role of socioeconomic disparities in modifying these associations: drought conditions were associated with greater risk in less urbanized regions, while excessive rainfall disproportionately impacted highly urbanized areas. These results offer critical insights into the interaction between climate and socioeconomic factors in disease dynamics, providing robust evidence to inform targeted surveillance and prevention strategies.

**Implications of all the available evidences**

This study underscores the importance of integrating climatic and socioeconomic dimensions into public health surveillance and policy development for SFTS. Addressing regional disparities and tailoring interventions based on local contexts can enhance early warning systems and reduce the disease burden, particularly among vulnerable populations. Moreover, our findings highlight the necessity of adapting public health strategies to the dual challenges of urbanization and extreme weather events in the era of global climate change. By prioritizing data-driven, region-specific interventions, policymakers can better mitigate the risks associated with SFTS and other climate-sensitive diseases.

## Introduction

Severe fever with thrombocytopenia syndrome (SFTS) is an emerging tick-borne viral hemorrhagic fever caused by the SFTS virus (SFTSV), also known as *Bandavirus dabieense*.^1^^,^^2^ Initially reported in China in 2010, SFTS has since been identified in other Asian countries, such as Korea, Japan, Vietnam, and Myanmar.^3^, ^4^, ^5^, ^6^, ^7^ Given its expanding distribution and high case fatality rate,^8^ the World Health Organization included SFTS as a research prioritization in 2017.^9^ Humans are primarily infected with SFTSV through tick bites or exposure to the blood or body fluids of infected individuals. *Haemaphysalis longicornis* (*H. longicornis*) ticks are recognized as the primary vector for SFTSV,^10^ although other tick species may also contribute to maintaining the virus in endemic regions. Native to Southeast Asia, *H. longicornis* has since 2017 been reported in 17 states in the United States (U.S.) and been found in New Zealand and Australia.^11^, ^12^, ^13^ The expansion of *H. longicornis*'s geographical range is believed to be facilitated by bird-mediated transportation over long distances, thereby introducing SFTSV into previously unaffected regions. Recent dissemination of SFTSV infection in China has been reported, with human cases of SFTS predicted to reach as far north as Jilin, Heilongjiang, and Inner Mongolia provinces with future climate change.^14^

The spatial–temporal distribution and spread of SFTS, primarily driven by tick vectors, are influenced by a complex interaction of ecological, environmental, and socioeconomic factors affecting human exposure to SFTSV-positive *H. longicornis*.^14^ The distinct seasonal and spatial clustering patterns of SFTS indicate common natural and social characteristics that contribute to its high incidence.^15^ Although meteorological factors have been extensively investigated for their impact on SFTS occurrence, these investigations have mainly focused on annual variations at the provincial level. For example, a study conducted in Shandong Province revealed a non-linear inverted “U”-shaped correlation between SFTS incidence and both average annual temperature and precipitation using generalized additive models, with inflexion points at approximately 12.5–13.0 °C for temperature and 650 mm for precipitation.^16^ Similarly, Sun et al. found an inverted “V”-shaped response curve for mean annual temperature (12.5–17.5 °C), cumulative precipitation (700–2250 mm), and mean relative humidity (63–82%) in Zhejiang province.^17^ Another study conducted in Jiangsu Province revealed that the maximum temperature in the warmest month, within an optimal range of 32.8–34.2 °C, had the most significant impact on the annual SFTS incidence.^18^

However, previous studies primarily focused on fundamental aspects such as habitat, landscape, and climate factors associated with the ecoepidemiology of SFTS. Nevertheless, they lacked an in-depth investigation into the interactions and synergistic effects of these factors. In China, SFTS cases have been reported across various administrative regions characterized by climatic and landscape compositions. Therefore, an integrated study that comprehensively examines all contributing factors within high-incidence areas nationwide is imperative. Furthermore, recent climate changes driven by global warming have led to extreme weather events such as heatwaves, floods and deep freezes, which directly or indirectly impact human and animal habitats, while also modifying anthropogenic factors that affect patterns of human disease transmission.^19^^,^^20^ Further investigation is still required to understand the impact of extraordinary meteorological conditions on SFTS incidence.

In this study, we employed hierarchical Bayesian spatiotemporal models in conjunction with distributed lag non-linear models (DLNMs) to investigate the nonlinear and lag-dependent associations between SFTS notification rate and climatic factors. Furthermore, we explored how these relationships are modified by levels of urban development under different socioeconomic conditions.

## Methods

### Data source and study area

Data on monthly laboratory and clinical confirmed SFTS cases between January 1st 2011 and December 31st 2022 were obtained for each county from the China Information System for Disease Control and Prevention (CISDCP). This system is an Internet-based real-time disease-reporting system reporting notifiable diseases that covers 55,077 national health facilities in 397 cities of all 31 provinces in the mainland of China.^21^ Standardized data of individual cases are electronically transmitted from hospitals, local Centers for Disease Control and Prevention (CDCs), community health centers, township health centers, and village clinics to the central database located at the China CDC. All notifiable infectious diseases were diagnosed according to their standard diagnostic criteria. Data uploaded by hospitals will be further verified by staff at local CDC for mistakes, omissions, duplications, and laboratory test. The notification rate of SFTS at the county level was calculated by dividing the number of cases by the annual population.

Monthly meteorological indicators, including mean temperature (°C), maximum temperature (°C), minimum temperature (°C), and average precipitation (mm), and were obtained from 890 weather surveillance stations across China (Figure S1). These data were recorded by the China Meteorological Data Service Center (https://data.cma.cn/). Gridded climate data with a 1 km × 1 km resolution were generated using ANUSPLIN-SPLINA software, with elevation as the independent covariate.^22^ To characterize drought and wet conditions, we employed the Standardized Precipitation Evapotranspiration Index (SPEI), based on deviations from average precipitation and potential evapotranspiration difference (Appendix page 3–5). According to the national standards of the People's Republic of China “Grades of Meteorological Drought (GB/T 20481-2017)”,^23^ an SPEI ≤ −0.5 indicates drought conditions, while an SPEI ≥ 0.5 indicates wet conditions in the study area (Table S2). Hydrological conditions were assessed across multiple timescales^24^ using SPEI-1, SPEI-3, SPEI-6, and SPEI-12 to assess hydrological conditions over 1, 3, 6, and 12 months, respectively.

According to the Chinese national standard (GB/T 40482-2021),^25^ five key socioeconomic indicators at the county level were used to comprehensively assess urban development: the gross domestic product (GDP) per capita, proportion of value-added of primary industry (agriculture, forestry, animal husbandry, and fisheries sectors),^26^ number of medical institutions (general hospitals, specialty hospitals, community health centers, clinics, and emergency medical centers) per capita, and proportion of urban population were obtained from county statistical yearbooks (Figure S2). The proportion of urban construction land was derived from gridded data on land use with a resolution of 1 km × 1 km (Institute of Geographic Sciences and Natural Resources Research, Chinese Academy of Sciences, http://data.ess.tsinghua.edu.cn/data/Simulation/).

Based on the annual notification rate of SFTS from 2011 to 2022, we initially identified the five provinces with the highest rate. Based on our previous research that applied a spatial trend surface analysis, four graphical clusters of SFTS cases in China were identified.^15^ Within these four clusters, we then compared the effects of temperature, precipitation, and hydrological status on SFTS notification rate.

### The modeling analysis

To address potential overdispersion in SFTS case counts, a negative binomial distribution was applied. A hierarchical Bayesian spatiotemporal model incorporating DLNMs was used to assess the association between monthly SFTS notification rate and temperature, precipitation, and SPEI over a period of up to 6 months. Natural cubic splines with two equal spaced internal knots for exposure and one equal knot for lag dimensions (on a log scale) were employed in the cross-basis function linking SFTS notification rate with meteorological factors. We calculated two key metrics: (1) lag-specific relative risk (*RR*) represents the risk ratio at each individual lag period (e.g., 0, 1, 2 months), reflecting the immediate impact of meteorological factors at each lag; (2) cumulative *RR* over a 6-month period (0–6 months) aggregates the effects across all lags, providing a comprehensive measure of the total influence of meteorological factors on SFTS risk over time. Model parameters were estimated using integrated nested Laplace approximations (INLA)^27^ within a Bayesian framework implemented in R version 4.2.2.

The effects of temperature, precipitation, and hydrological conditions on the seasonal dynamics of SFTS were examined using a four-step modeling approach. This model sequentially introduced random effects, extreme weather variables, temperature, and precipitation. At each step, the optimal model was selected based on the minimum deviance information criterion (DIC). Firstly, a Baseline model was constructed with province-level monthly autocorrelated random effects (Figure S3) and annual county-level spatial random effects (Figure S4). Secondly, a Baseline-SPEI model was developed by sequentially introducing four SPEI variables (SPEI-1, SPEI-3, SPEI-6, and SPEI-12) to achieve the best goodness of fit. In the third step, a Baseline-SPEI-TEMP model was established by further incorporating three monthly temperature variables (maximum, minimum, and mean temperature). Finally, a Baseline-SPEI-TEMP-PREC model was created by adding average precipitation, and the optimal models were chosen based on the smallest DIC. The improved model fits were assessed at each step by comparing mean absolute error (MAE) with that of the baseline models to identify counties that benefitted from the more complex models.

The model's predictive performance was assessed using a leave-one-out cross-validation. This process involved simulating posterior predictive distributions of SFTS cases by sampling from the model's posterior distributions of parameters and hyperparameters. The process was repeated 12 × 12 times, each time excluding one month per year. Finally, observed values were compared with the out-of-sample posterior predictive distributions for each province from January 2011 to December 2022.

### Sensitivity analyses

To assess the robustness of the model results, sensitivity analyses were conducted by varying the number of knots in the exposure and lag dimensions of the cross-basis functions for meteorological and hydrological indicators. Additionally, we assessed the impact of underreporting by increasing reported SFTS cases by 10%–30% and examined potential COVID-19 disruptions by excluding 2020–2022 from the analysis.

### Assessment of modifying effects

To examine the modifying effects of socioeconomic conditions, standardized linear interaction terms between five key socioeconomic indicators and temperature, precipitation, and hydrological variables were included in the final model across all counties in the study areas.

### Ethics approval

Written informed consent was waived by the National Health Commission of China for the surveillance of notifiable infectious disease. All the data of cases used in this study were anonymized and personal identification information is not included in the data. This study was approved by the Institutional Review Boards of the Academy of Military Medical Science (IRB number: AF/SC-08/02.343).

### Role of the funding source

The funders played no role in study design, data collection, data analysis, data interpretation, or manuscript writing.

## Results

### Spatiotemporal pattern of SFTS cases

**Fig. 1:**
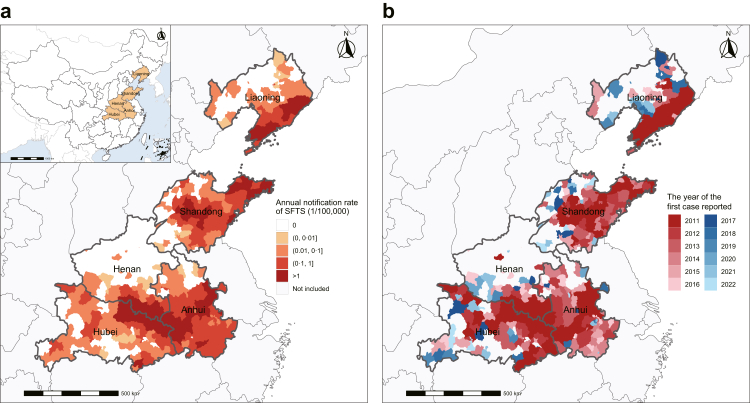
**Geographic locations and spatial distribution of SFTS notification rate by county in the five highest-notification rate provinces of China from 2011 to 2022**. (a) Annual notification rate of SFTS (1/100,000) from 2011 to 2022. (b) The spatial dynamics of SFTS from 2011 to 2022. Colors in panel B represent the first year in this range in which cases were reported.

Based on the annual notification rate from January 2011 to December 2022, Anhui had the highest annual notification rate of SFTS (0.522/10^5^), followed by Shandong (0.521/10^5^), Henan (0.431/10^5^), Hubei (0.376/10^5^), Liaoning (0.203/10^5^), Zhejiang (0.131/10^5^), and Jiangsu (0.074/10^5^). The top five provinces with the highest SFTS notification rate were included (Anhui, Shandong, Henan, Hubei, and Henan). A total of 20,696 confirmed SFTS cases were reported in 59.6% (360/604) of the counties within these provinces, accounting for 92.4% of all cases nationwide (20,696/22,398) (Fig. 1a).

A geographic expansion of SFTS cases was observed, with the number of affected counties increasing from 104 in 2011 to 360 in 2022 (Fig. 1b). Henan Province showed a peak notification rate in 2015, followed by a decline and another surge starting from 2020. The other four provinces demonstrated a steady increase over the years, with a marked rise starting from 2020 (Figure S5A). An obvious seasonal pattern was observed, with peak notification rate occurring between May and October; however, the time of peak varied by latitude. For instance, Liaoning Province (higher latitude) peaked in July, compared to June in Shandong and May in Henan, Anhui, and Hubei (Figure S5B). This seasonal heterogeneity might be associated with varying meteorological and hydrological conditions across the five provinces (Figures S6‒S7).

### Modeling process and evaluation of the model

By applying a four-step modelling process, we obtained the optimal Baseline-SPEI model incorporating SPEI-1 (DIC = 41,793), the optimal Baseline-SPEI-TEM model incorporating monthly maximum temperature (DIC = 41,325), and the optimal Baseline-SPEI-TEMP-PREC model incorporating average precipitation (DIC = 41,059); The final model achieved a minimum DIC of 41,059 by incorporating SPEI-1, maximum temperature and precipitation as predictive variables associated with SFTS notification rate (process detailed in Table S3).

The final model demonstrated an improved fit in 87.42% (528/604) of counties studied, as indicated by reduced MAE compared to the Baseline model (Figure S8). Among individual provinces, Henan showed the best performance, with improved fit in 97.48% (155/159) of counties. Liaoning followed with 94% (94/100%) of counties showing improvement, Hubei with 86.41% (89/103), Shandong with 86.13% (118/137), and Anhui with 68.58% (72/105). In counties where the baseline model performed better than the final model, it was likely that unrecognized factors influenced spatiotemporal dynamics.

**Fig. 2:**
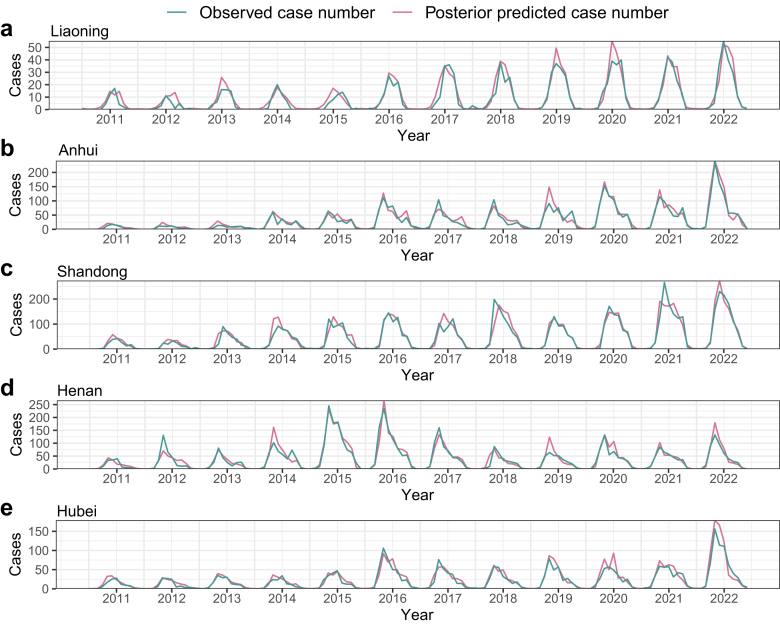
**The observed versus posterior predictive number of SFTS cases rates per province**. (a) Liaoning, (b) Anhui, (c) Shandong, (d) Henan, and (e) Hubei. The mean observed number of SFTS cases (green curve) and corresponding posterior predictive mean number of SFTS cases (solid pink curve) from January 2011 to December 2022 was estimated by Bayesian spatiotemporal models. SFTS, severe fever with thrombocytopenia syndrome.

The out-of-sample posterior predictive estimates for SFTS notification rate simulated from the cross-validation fitting of the Baseline-SPEI-TEMP-PREC model are summarized annually (Figure S9). The cross-validation demonstrated that the predicted cases number closely matched the reported counts during the study period (Table S4). Notably, the model accurately predicted the outbreaks in Anhui and Hubei in 2022, as well as the significant decline in SFTS cases observed in Henan since 2016 (Fig. 2). These results indicate robust model performance in simulating SFTS case counts and effectively capturing annual fluctuations.

### Effects of temperature, precipitation, and hydrological conditions on SFTS in five high-notification rate provinces

**Fig. 3:**
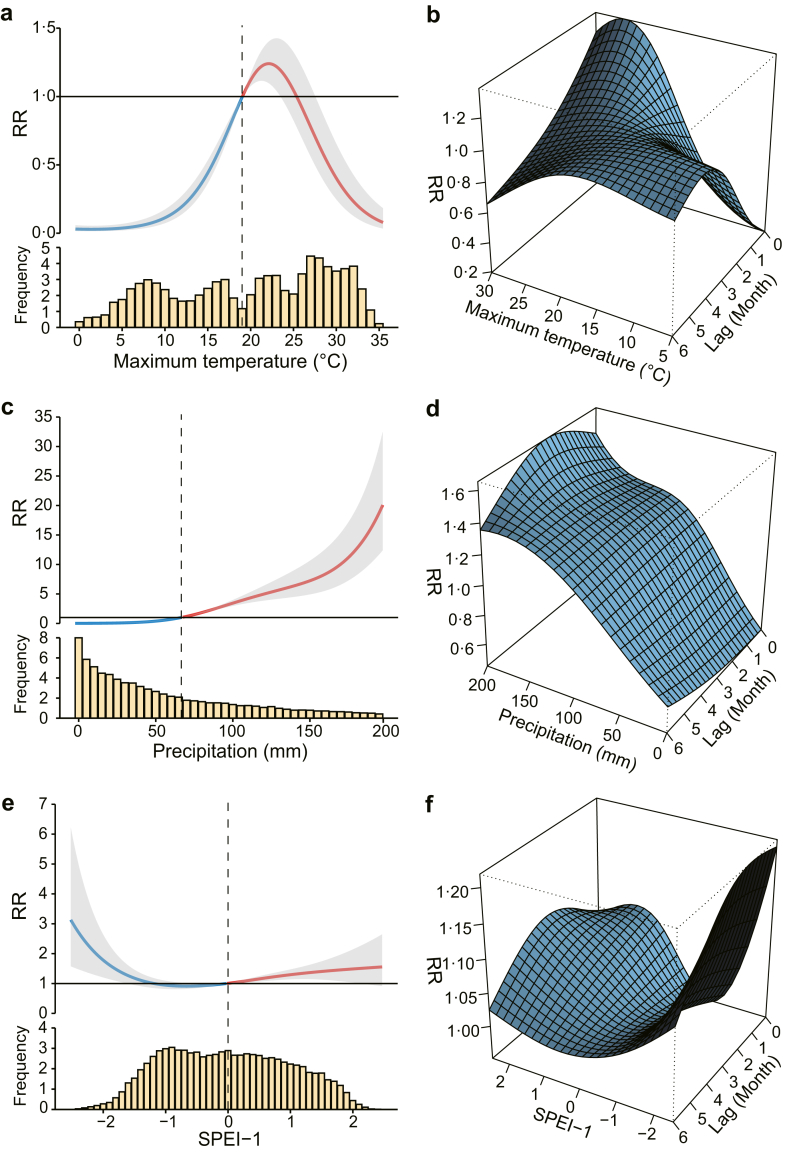
**Non-linear and lag-dependent effects of maximum temperature, mean precipitation, and SPEI-1 on the seasonal dynamics of SFTS notification rate**. (a–b) Cumulative exposure response effect and 3D map of maximum temperature on the risk of SFTS. (c–d) Cumulative exposure response effect and 3D map of precipitation on the risk of SFTS. (e–f) Cumulative exposure response effect and 3D map of SPEI-1 on the risk of SFTS. The vertical dashed lines in panel A, C, and E represent the reference values. The reference values for maximum temperature and precipitation are their respective medians, while the reference value for SPEI-1 is 0. The yellow bar in panel A, C, and E represent the frequency (in thousands). The gray shading represents the 95% confidence interval. SFTS, severe fever with thrombocytopenia syndrome; RR, relative risk; SPEI, Standardized Precipitation Evapotranspiration Index.

We observed heterogeneous effects of temperature, precipitation, and hydrological conditions on SFTS (Fig. 3). The impact of monthly maximum temperature on SFTS notification rate exhibited a non-linear, inverted “U” shaped relationship (Fig. 3a and b). The highest cumulative relative risk (*RR* = 1.24; 95% CI: 1.10–1.40) was observed at a temperature of 21.97 °C with a lag period of 0–6 months. When the monthly maximum temperature fell outside the threshold range of 18.99° C‒25.17 °C, the cumulative relative risk for SFTS significantly decreased to below 1. The month-specific effect of maximum temperature varied across different lag periods from 0 to 6 months, with stronger associations observed at shorter lags. Notably, both the highest and lowest relative risks for SFTS occurred at a lag of 0 months, corresponding to temperatures of 25.67 °C (*RR* = 1.38, 95% CI: 1.26–1.51) and 5 °C (*RR* = 0.19, 95% CI: 0.16–0.24), respectively (Fig. 3b, Table S5).

The risk of SFTS demonstrated a non-linear, upward-trending relationship with monthly precipitation levels, exhibiting a notably delayed response (Fig. 3c and d). When monthly precipitation was below 70 mm, the risk of SFTS remained below 1; however, it rose significantly at higher precipitation levels, with the cumulative *RR* peaking at 20.08 (95% CI: 12.40–32.54). Additionally, for the same level of precipitation, the impact on SFTS notification rate initially increased and then diminished over a lag period of 0–6 months as the lag duration lengthened, with the month-specific effect (*RR* = 1.65, 95% CI: 1.50–1.82). Notably, the month-specific effect peaked at a three-month lag when the precipitation reached 200 mm (Table S6).

The risk of SFTS exhibited a “U”-shaped response to SPEI-1 (Fig. 3e and f). Both drought and wet conditions increased the risk of SFTS compared to normal hydrological conditions. The highest cumulative *RR* was observed at an SPEI-1 value of −2.5 within a lag period of 0–6 months, with an *RR* of 3.13 (95% CI: 1.58–6.23) for drought conditions, and at an SPEI-1 value of 2.16 within a lag period of 0–6 months, with an *RR* of 1.51 (95% CI: 1.00–2.27) for wet conditions. For the month-specific effect, both drought and wet conditions significantly increased the risk of SFTS, within a lag period of 0–4 months and 0–1 month, respectively (Tables S7, S8). The highest risk was observed at an SPEI-1 of −2.5 with a 2-month lag (*RR* = 1.22, 95% CI: 1.08–1.37), and at an SPEI-1 of 1.81 with a 1-month lag (*RR* = 1.07, 95% CI: 1.02–1.13, respectively.

The sensitivity analyses demonstrated the model's robustness as minimal impact was observed when altering the number of knots in either the exposure or lag dimensions (Figure S10). Similarly, the associations remained consistent when accounting for potential underreporting (Figure S11) and when excluding the COVID-19 pandemic years (Figure S12).

### Effects of temperature, precipitation, and hydrological conditions on SFTS in different geographic clusters

Considering the diverse seasonal patterns observed across different geographical clusters^15^ (Figure S13), separate investigations of the impact of meteorological factors in four distinct geographic clusters were conducted. Precipitation and temperature consistently influenced SFTS notification rate across all four geographical clusters (Tables S9‒S10, Figures S14‒S15). In Clusters I and IV, the risk of SFTS followed an inverted “U”-shaped pattern relationship with the minimum temperature levels and was significantly associated with higher levels of precipitation compared to their respective medians. On the other hand, in Cluster III, the risk of SFTS was associated with higher mean temperature levels, which exhibited a triphasic decline-rise-decline pattern within a span of 6 months.

Hydrological factors had similar associations with SFTS in Clusters III and IV, which differed from Clusters I and II. The relationship between SPEI-12 and the risk of SFTS exhibited a comparable inverted “U”-shaped pattern in both clusters. Both drought and wet conditions increased the risk of SFTS within 6 months in these two clusters compared to normal hydrological conditions. Conversely, under drought conditions in Cluster II, there was an initial increase followed by a subsequent decrease in the risk of SFTS with increasing values of SPEI-12. Under wet conditions, there was an initial decrease followed by an increase in the risk of SFTS which peaked at SPEI-12 value of 1.5 with a *RR* of 5.66 (95% CI: 3.39–9.45).

### Modification effect of socioeconomic conditions

**Fig. 4:**
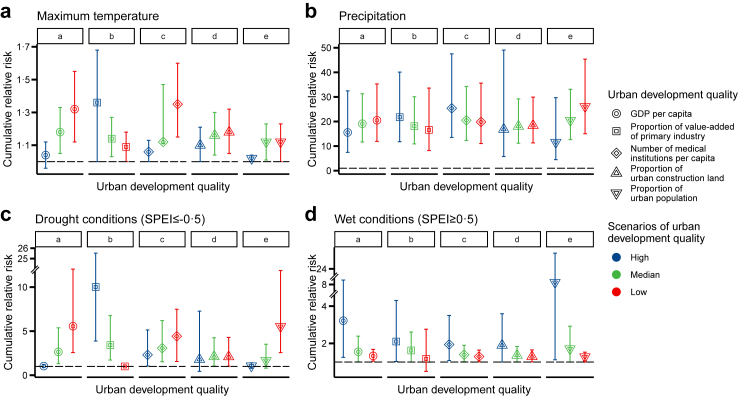
**Overview of the modification effects of socioeconomic factors on the association between meteorological indicators and the risk of SFTS**. (a) Maximum temperature, (b) Precipitation, (c) Drought conditions, (d) Wet conditions. The points indicate the relative risk of SFTS and error bars are defined as 95% confidence interval. Each panel includes five socioeconomic strata, labeled as “a” through “e” along the x-axis, corresponding to: a, GDP per capita; b, Proportion of value-added of primary industry; c, Number of medical institutions per capita; d, Proportion of urban construction land; e, Proportion of urban population. GDP, gross domestic product; SPEI, Standardized Precipitation Evapotranspiration Index.

Incorporating interactions between climatic and socioeconomic factors improved the model fit (Table S11). Socioeconomic factors played diverse roles in modifying the association between SFTS notification rate and temperature, precipitation, and hydrological conditions (Fig. 4). The impacts of maximum temperature and precipitation on the risk of SFTS were found to be more pronounced in low-quality urban development scenarios compared to high-quality ones. Specifically, drought conditions were associated with an increased risk of SFTS in low-quality urban development scenarios, while wet conditions heightened the risk in high-quality urban development scenarios (Table 1, Tables S12‒S15).

**Table 1:** Maximum month-specific and cumulative relative risk of SFTS for meteorological factors within 6 months by different scenarios of GDP per capita.

Meteorological factor	GDP per capita	Maximum *RR*			Maximum cumulative *RR*	
		Value	Lag (month)	*RR* (95% CI)	Value	*RR* (95% CI)
Maximum temperature	High	7.3 °C	6	1.23 (1.01‒1.50)	20.4 °C	1.04 (0.96–1.12)
	Median	25.9 °C	0	1.38 (1.25–1.52)	21.8 °C	1.18 (1.05–1.33)
	Low	26.8 °C	0	1.51 (1.34–1.70)	22.6 °C	1.32 (1.12–1.55)
Precipitation	High	200 mm	2	1.66 (1.49–1.85)	200 mm	15.55 (7.45–32.48)
	Median	200 mm	3	1.65 (1.49–1.82)	200 mm	19.12 (11.69–31.27)
	Low	200 mm	3	1.65 (1.49–1.84)	200 mm	20.51 (11.92–35.29)
SPEI-1 at drought conditions (SPEI-1≤−0.5)	High	−0.9	5	1.04 (1.00–1.07)	−0.7	1.04 (0.86–1.26)
	Median	−2.5	2	1.21 (1.07–1.37)	−2.5	2.65 (1.31–5.38)
	Low	−2.5	2	1.36 (1.18–1.57)	−2.5	5.56 (2.57–12.04)
SPEI-1 at wet conditions (SPEI-1 ≥0.5)	High	2.5	3	1.30 (1.08–1.55)	2.5	3.22 (1.25–8.30)
	Median	2.3	2	1.09 (1.00–1.18)	2.2	1.55 (1.01–2.39)
	Low	1.3	0	1.06 (1.01–1.12)	1.4	1.33 (1.05–1.68)

SFTS, severe fever with thrombocytopenia syndrome; RR, relative risk; CI, confidence interval; SPEI, Standardized Precipitation Evapotranspiration Index; GDP, gross domestic product.

The different levels of GDP per capita were obtained by centralizing each county to the 25th, 50th, and 75th percentiles.

The impacts of SPEI on the risk of SFTS are presented under drought and wet conditions, respectively.

The classification of weather conditions was based on the national standards of the People's Republic of China “Grades of Meteorological Drought (GB/T20481-2017)”.

The reference groups were the median monthly maximum temperature (19 °C), the median monthly precipitation (70 mm), and an SPEI value of 0 for normal hydrological conditions.

**Fig. 5:**
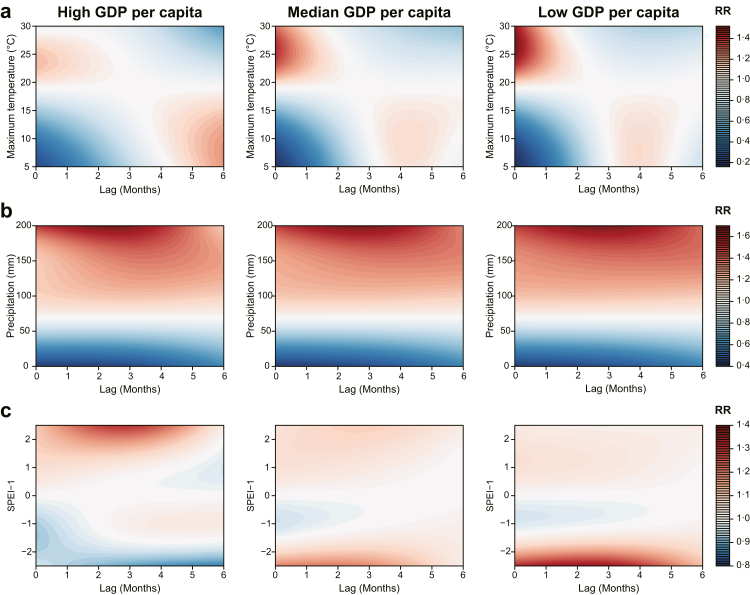
**Modification effect of GDP per capita on the association between SFTS notification rate and meteorological factors**. (a) Maximum temperature, (b) Precipitation, (c) SPEI-1. Scenarios with a high GDP per capita, medium GDP per capita, and low GDP per capita were demonstrated. The GDP per capita in the interaction term was centered on its 25th, 50th, and 75th percentile of the 604 county's value range. GDP, gross domestic product; RR, relative risk; SPEI, Standardized Precipitation Evapotranspiration Index.

The modification effects of five socioeconomic factors were presented separately (Fig. 5, Figures S16‒S19). For instance, GDP per capita demonstrated a modifying effect on the association between SFTS and all three meteorological factors (Fig. 5). A positive association with maximum temperature was observed, with the highest effect occurring under low GDP per capita condition. Specifically, at a maximum temperature of 26.8 °C with no lag, the *RR* was estimated to be 1.51 (95% CI: 1.34–1.70). In contrast, under high GDP per capita scenario, a lower effect was observed (*RR* = 1.23, 95% CI: 1.01‒1.50), at a maximum temperature of 7.3 °C with a lag of 6 months. Similarly for cumulative effects within a period of 0–6 months, higher effects associated with maximum temperature were demonstrated under low GDP per capita; the maximum cumulative *RR* for high-, medium-, and low-GDP scenarios were estimated as 1.04 (95% CI: 0.96–1.12), 1.18 (95% CI: 1.05–1.33), and 1.32 (95% CI: 1.12–1.55), respectively (Table 1).

Similarly, the positive association between SFTS and precipitation attained its peak during a 0‒6-month period under low GDP per capita. The cumulative *RR* increased from 15.55 (7.45–32.48) for high GDP to 19.12 (11.69–31.27) for medium GDP and 20.51 (11.92–35.29) for low GDP, respectively (Table 1). The association between SFTS and hydrological conditions was also modified by GDP per capita. Under low GDP levels, the risk of SFTS notification rate increased during drought conditions (maximum *RR* of 1.36; 95% CI 1.18–1.57 observed at SPEI-1 of −2.5, with a lag of 2 months). However, under high GDP levels, the effect was smaller (maximum *RR* of 1.04; 95% CI 1.00–1.07 at SPEI-1 of −0.9, with a lag of 5 months). During drought periods, the impact of SPEI-1 on the risk decreased as GDP per capita increased, but increased as it decreased in value. The cumulative risk peaked at 5.56 (2.57–12.04) under low GDP per capita and SPEI-1 = −2.5 (Table 1). By contrast, during wet conditions, the effect of SPEI-1 on SFTS notification rate decreased under low GDP levels (maximum *RR* of 1.06; 95% CI 1.01‒1.12 at an SPEI-1 of 1.3 with no lag), which was lower than the effect observed under high GDP per capita (maximum *RR* of 1.30, 95% CI 1.08–1.55 at an SPEI-1 of 2.5 with a lag of 3 months (Table 1).

## Discussion

In this study, we quantified the nonlinear and lagged impacts of temperature, precipitation, and hydrological conditions on SFTS incidence, providing insights into the seasonal dynamics and heterogeneous patterns across provinces and geographic clusters. A modification effect by multidimensional socioeconomic factors was identified. Both drought and wet conditions increased the risk of SFTS compared to normal hydrological conditions; however, this effect was modulated by socioeconomic factors. The results demonstrated a nonlinear relationship between monthly temperature and the risk of SFTS, characterized by a lagged and inverted “U”-shape. Sun et al.’s findings align with ours, as they also reported an inverted “U”-shaped non-linear association between weekly average temperature and SFTS incidence.^28^ The causal explanations for these correlations can be substantiated from both biological and sociological perspectives, primarily concerning tick survival, reproduction, and host-seeking behavior.^29^ For example, ticks exhibit increased metabolic rates, accelerated growth, and enhanced reproductive capacity under tolerable high temperatures, which contributed to population expansion and increased opportunities for virus transmission to natural hosts.^30^ Elevated temperatures may also extend the geographic range of *H. longicornis* by accelerating its life cycle, while sustained humidity facilitates viral persistence in tick saliva.^31^ Temperature future affects host behavior and ecological traits.^32^ In addition, temperature influences the migratory patterns of birds, which are known to facilitate the long-distance dispersal of *H. longicornis* and SFTSV. Studies have shown that bird migration routes align with the geographic distribution of SFTSV, suggesting that climate-driven shifts in avian migration may contribute to the spread of both the tick vector and the virus.^5^^,^^33^ The relationships between hydrological conditions and the risk of SFTS were consistent in Clusters III and IV, aligning with the observed effect patterns when considering all cases. Both clusters share similar ecological characteristics, centered around mountainous areas (Mount Tai and Mount Dabie, respectively), located far from the coastline. In contrast, the exposure-lag-response association in Clusters I and II differed from the other two clusters, potentially related to their location in peninsular regions where the interaction between oceanic and terrestrial hydrological cycles modulates local meteorological and hydrological conditions. The distinct effects observed between Clusters III/IV (Mountain type) and Clusters I/II (Peninsular type) underscore the importance of tailoring disease surveillance and prevention measures to local environmental conditions.

For the first time, we have identified how urban development quality affects the association between SFTS notification rate and meteorological factors, as well as hydrological conditions. In areas with low-quality urban development, an elevated risk of SFTS is associated with higher maximum temperatures. Inferior urban development often entails inadequate infrastructure, such as insufficiently developed air conditioning systems, ventilation facilities, and thermal insulation within residential areas. Additionally, low-quality economic development may rely heavily on labor-intensive industries that predominantly involve outdoor work. This results in prolonged outdoor exposure for a larger population, thereby increasing the likelihood of contact with ticks and reservoir animals carrying SFTSV.^34^

Similarly, in areas characterized by low urban development quality, drought conditions significantly heightened the risk of SFTS compared to regions with high urban development quality. This can be attributed to the increased frequency of human interaction with ticks or animal hosts carrying SFTSV in agriculturally dominated regions.^35^ Drought conditions result in sparse vegetation and alterations in animal husbandry practices, leading reservoir animals and tick vectors to congregate around limited water sources and even migrate toward human settlements in search of water. Consequently, there are greater opportunities for humans to come into contact with SFTSV carried by these reservoir animals and vectors.^36^ For instance, a study in Kenya found that calves had a higher tick burden during the dry season.^37^ Conversely, in areas with low urban development quality, wet conditions were associated with a reduced risk of SFTS compared to regions with high urban development quality. While excessive precipitation can create favorable conditions for tick proliferation and increase the occurrence of SFTSV,^38^ extreme wet conditions may decrease the risk of SFTS in rural areas. Flooding events could disrupt tick habitats and reduce outdoor agricultural activities among locals, thereby lowering the probability of human exposure to ticks carrying SFTSV. This highlights the intricate ecological balance between humans and the natural environment, leading to diverse consequences across rural and urban regions.

While our study primarily focused on the associations between meteorological, hydrological, and socioeconomic factors with SFTS incidence, we recognize the importance of human behavior, such as outdoor activity, in modulating disease risk. As demonstrated in Yoo et al.’ study, SFTSV-infected ticks were present during winter in South Korea, yet no human cases were observed. This suggests that reduced outdoor activities during colder months may contribute to lower transmission risk.^39^ Moreover, our findings indirectly support the relationship between outdoor activity and SFTS incidence. For instance, the observed seasonality of SFTS cases—with peaks during warmer months and an absence in winter—correlates with periods of increased outdoor activity, particularly in rural and agricultural settings where individuals are more likely to encounter tick habitats. Additionally, our results suggest that urban development quality modifies the impact of climate son SFTS risk, which may partially reflect differences in occupational and recreational exposure to ticks.

The limitations of this study should be acknowledged. Firstly, the potential underestimation of SFTS cases due to inadequate diagnostic methods and disease surveillance systems in rural and remote areas, might introduce bias in case classification. Underreporting remains a concern, particularly in regions with limited healthcare access and surveillance capacity, which may impact the completeness of the dataset. Secondly, due to the lacking of nation-wide tick surveillance data, the direct effects of climatic factors on tick density and their subsequent impact on SFTS risk cannot be explored. Future studies that incorporate detailed tick density data are warranted to better elucidate the correlation between climatic factors, tick density, and SFTS incidences. Similarly, the absence of migratory bird monitoring data prevents direct assessment of their potential role in the long-distance dispersal of *H. longicornis* and SFTSV transmission. Additionally, outdoor activity patterns, which influence human exposure to tick habitats, were not accounted for due to the lack of region-specific behavioral data, potentially limiting the ability to fully capture seasonal transmission dynamics. Future research is needed to better understand how behavioral patterns interact with environmental and socioeconomic factors to shape SFTS risk.

This study establishes an epidemiological association between the seasonal dynamics of SFTS and hydrometeorological conditions, demonstrating an increased risk of SFTS associated with elevated temperature, increased precipitation, as well as drought, and wet climate conditions, with varying lag effects observed. These findings underscore the necessity of integrating climatic factors into SFTS surveillance and early warning systems, particularly by incorporating specific climatic thresholds such as SPEI ≤ −1.5 to improve risk prediction accuracy. For practical implementation, real-time meteorological data and climate models can be embedded within existing disease surveillance frameworks to provide outbreak warnings for high-incidence seasons, facilitating proactive interventions. Additionally, spatial risk mapping based on these climate indicators can assist in identifying vulnerable areas and optimizing guide resource allocation for vector control and public health preparedness.

The study's findings can inform the establishment of actionable public health priorities by highlighting the importance of active surveillance programs and targeted educational campaigns. While climatic factors are non-modifiable, enhanced and tailored interventions at the human–environment interface can effectively mitigate disease risks. For instance, policymakers could develop and implement more targeted and adaptive surveillance systems that account for seasonal and spatial variations in disease risk. Additionally, intervention strategies could emphasize public health initiatives to educate communities about heightened risks during specific climatic conditions, as well as the implementation of measures (such as tick control and vegetation management) to address environmental factors exacerbating disease transmission, particularly among high-risk populations involved in outdoor activities.

## Contributors

Hong-Han Ge: conceptualization, data curation, formal analysis, investigation, methodology, software, visualization, writing—original draft, Kun Liu: investigation, methodology, writing—review & editing. Fang-Yu Ding: investigation, methodology, funding acquisition, writing—review & editing, Peng Huang: investigation, writing—review & editing. Yan-Qun Sun: investigation, writing—review & editing. Ming Yue: investigation, writing—review & editing. Hong Su: investigation, writing—review & editing. Qian Wang: investigation, writing—review & editing. Nicholas Philip John Day: investigation, writing—review & editing. Richard James Maude: investigation, writing—review & editing. Dong Jiang: investigation, writing—review & editing. Li-Qun Fang: investigation, data curation, project administration, resources, supervision, writing—review & editing. Wei Liu: investigation, data curation, funding acquisition, project administration, resources, supervision, writing—review & editing. Hong-Han Ge, Li-Qun Fang, and Wei Liu accessed and verified the data and had full access to all the raw data used in the study. All authors had final responsibility for the decision to submit the manuscript for publication.

## Data sharing statement

The datasets generated and/or analyzed during the current study are available from the corresponding author (WL) upon reasonable request.

## Editor note

The Lancet Group takes a neutral position with respect to territorial claims in published maps and institutional affiliations.

## Declaration of interests

The authors declare that there is no conflict of interests.
