# Supplementary material for: Combined effects of hydrological conditions and socioeconomic factors on the seasonal dynamics of severe fever with thrombocytopenia syndrome in China, 2011–2022: a modelling study
Source: Lancet Reg Health West Pac. 2025 Apr 28;58:101564. doi: 10.1016/j.lanwpc.2025.101564 (PMC12243062; doi:10.1016/j.lanwpc.2025.101564)
Supplement: Supplementary Figures and Tables [file mmc1.docx]

**Supplementary appendix**

Combined effects of hydrological conditions and socioeconomic factors on the seasonal dynamics of severe fever with thrombocytopenia syndrome in China, 2011‒2022: a modelling study.

**Contents**

[**Supplementary Material and Methods** 3](#_Toc195563889)

[**Supplementary References** 8](#_Toc195563890)

[**Table S1.** Explanation and source of variables used in this study. 9](#_Toc195563891)

[**Table S2.** Classification of weather conditions based on SPEI. 10](#_Toc195563892)

[**Table S3.** The performance of the fitted model in the four-step modeling approach. 11](#_Toc195563893)

[**Table S4.** The estimated numbers of SFTS case by province, and the differences in predicted number of cases compared with the reported number of cases from 2011-2022. 12](#_Toc195563894)

[**Table S5.** Maximum and minimum relative risk of SFTS associated with maximum temperature for each month within 0-6 months. 13](#_Toc195563895)

[**Table S6.** Maximum and minimum relative risk of SFTS associated with precipitation for each month within 0-6 months. 14](#_Toc195563896)

[**Table S7.** Maximum and minimum relative risk of SFTS associated with drought conditions (SPEI≤-0.5) for each month within 0-6 months. 15](#_Toc195563897)

[**Table S8.** Maximum and minimum relative risk of SFTS associated with wet conditions (SPEI≥0.5) for each month within 0-6 months. 16](#_Toc195563898)

[**Table S9.** The performance of the model fit in the four-step modeling approach on four geographical clusters with SFTS. 17](#_Toc195563899)

[**Table S10.** Maximum relative risk of SFTS for meteorological factor within 6 months. 19](#_Toc195563900)

[**Table S11.** The DIC of the model after incorporating the interaction terms between climatic and socioeconomic factors in the final model. 20](#_Toc195563901)

[**Table S12.** Maximum and cumulative relative risk of SFTS for meteorological factors within 6 months by different scenarios of proportion of value-added of primary industry. 21](#_Toc195563902)

[**Table S13.** Maximum and cumulative relative risk of SFTS for meteorological factors within 6 months by different scenarios of numbers of medical institutions per capita. 22](#_Toc195563903)

[**Table S14.** Maximum and cumulative relative risk of SFTS for meteorological factors within 6 months by different scenarios of proportion of urban construction land. 23](#_Toc195563904)

[**Table S15.** Maximum and cumulative relative risk of SFTS for meteorological factors within 6 months by different scenarios of proportion of urban population. 24](#_Toc195563905)

[**Figure S1.** Geographical distribution of 890 weather surveillance stations in China. 25](#_Toc195563906)

[**Figure S2.** Spatial distribution of annual mean value of socioeconomic factors for the 604 counties of the five high-incidence provinces in China from 2011-2022. 26](#_Toc195563907)

[**Figure S3.** Posterior distributions of province-specific autocorrelated monthly random effects. 27](#_Toc195563908)

[**Figure S4.** Contribution of year-specific spatial random effects to the number of SFTS cases estimates. 28](#_Toc195563909)

[**Figure S5.** The annual and monthly incidence rates of SFTS of the five high-incidence provinces from 2011 to 2022. 29](#_Toc195563910)

[**Figure S6.** Monthly maximum temperature, minimum temperature, mean temperature, and precipitation of the five high-incidence provinces from 2011 to 2022. 30](#_Toc195563911)

[**Figure S7.** Monthly SPEI-1, SPEI-3, SPEI-6, and SPEI-12 of the five high-incidence provinces from 2011 to 2022. 31](#_Toc195563912)

[**Figure S8.** Added value of using final model compared to the baseline model. 32](#_Toc195563913)

[**Figure S9.** Posterior predictive mean SFTS incidence rate from 2011-2022. 33](#_Toc195563914)

[**Figure S10.** Sensitivity results of relative risk of meteorological factors on the risk of SFTS. 34](#_Toc195563915)

[**Figure S11.** Sensitivity analysis of underreporting in SFTS notifications. 35](#_Toc195563916)

[**Figure S12.** Sensitivity analysis excluding the COVID-19 pandemic years. 36](#_Toc195563917)

[**Figure S13.** Spatial distribution of four geographic clusters of SFTS cases in China. 37](#_Toc195563918)

[**Figure S14.** 3D map of temperature, precipitation, and SPEI on the risk of SFTS in four geographical clusters. 38](#_Toc195563919)

[**Figure S15.** Cumulative exposure-response effect of temperature, precipitation, and SPEI on the risk of SFTS in four geographical clusters. 39](#_Toc195563920)

[**Figure S16.** Modification effects of proportion of value-added of primary industry on the association between SFTS incidence and meteorological factors. 40](#_Toc195563921)

[**Figure S17.** Modification effects of numbers of medical institutions per capita on the association between SFTS incidence and meteorological factors. 41](#_Toc195563922)

[**Figure S18.** Modification effects of proportion of urban construction land on the association between SFTS incidence and meteorological factors. 42](#_Toc195563923)

[**Figure S19.** Modification effects of proportion of urban population on the association between SFTS incidence and meteorological factors. 43](#_Toc195563924)

**Supplementary Material and Methods**

**Computation of Standardized Precipitation Evapotranspiration Index**

Numerous indices have been developed for monitoring hydrological conditions, including Palmer Drought Severity Index (PDSI), Standardized Precipitation Index (SPI), and Standardized Precipitation Evapotranspiration Index (SPEI). The SPI, however, relies exclusively on precipitation data and neglects the thermal effects on evaporation. While PDSI incorporates evapotranspiration processes, it is constrained by its dependence on soil moisture balance models. In contrast, SPEI directly quantifies water deficit/surplus states by computing the discrepancy between precipitation and potential evapotranspiration, thus providing a more mechanistic representation of environmental conditions relevant to SFTS transmission dynamics.

First, the Thornthwaite method was utilized to calculate the Potential Evapotranspiration Index (PEI)^1^. This method primarily relies on average temperature, with consideration of latitude information. The formula is as follows:

$$PET=16\left(\frac{10T_{i}}{H}\right)^{A}$$

$$H_{i}=\left(\frac{T_{i}}{5}\right)^{1.514}$$

$$H=\sum_{i=1}^{12} H_{i}=\sum_{i=1}^{12} \left(\frac{T_{i}}{5}\right)^{1.514}$$

where $T_{i}$represents the average temperature, $H_{i}$denotes the monthly heat index, and $H$stands for the annual heat index. Subsequently, $D_{i}$ is calculated based on the difference between precipitation and PET:

$$D_{i}=P_{i}-PET_{i}$$

$$\left\{\begin{matrix} D_{i,j}^{k}=\sum_{m=13-k+j}^{12} D_{i-1,m}+\sum_{m=1}^{j} D_{i,m} & j<k \\ D_{i,j}^{k}=\sum_{m=j-k+1}^{j} D_{i,m} & j\geq k \end{matrix}\right.$$

where $D_{i,j}^{k}$denotes the accumulated PET within $k$ months starting from the $i$th month of the $j$th year. Subsequently, a three-parameter log-logistic distribution is fitted to the numerical sequence $D_{i}$, expressed as:

$$F\left(x\right)=\left[1+\left(\frac{\alpha}{\gamma}\right)^{\beta}\right]^{-1}$$

The parameters $\alpha$, $\beta$, and $\gamma$in the formula are calculated using the L-moment estimation method:

$$\left\{\begin{matrix} \alpha=\frac{\left(w_{0}+2w_{1}\right)_{\beta}}{\Gamma\left(1+\frac{1}{\beta}\right)\Gamma\left(1-\frac{1}{\beta}\right)} \\ \beta=\frac{2w_{1}-w_{0}}{6w_{1}-w_{0}+6w_{2}} \\ \gamma=w_{0}-\alpha\Gamma\left(1+\frac{1}{\beta}\right)\Gamma\left(1-\frac{1}{\beta}\right) \end{matrix}\right.$$

$$w_{s}=\frac{1}{M}\sum_{i=1}^{M} \left(1-F_{i}\right)^{s}D_{i}$$

$$F_{i}=\frac{i-0.35}{M}$$

where $\Gamma\left(\beta\right)$ denotes the Gamma function related to $\beta$, while $w_{1}$, $w_{2}$, and $w_{3}$ are the probability-weighted moments of $D_{i}$.$M$ represents the number of months, and $F_{i}$ is the frequency estimation value. The probability value of the normalized distribution function is calculated as follows:

$$P=1-F\left(x\right)$$

Finally, the Standardized Precipitation Evapotranspiration Index (SPEI) value can be calculated:

$$SPEI=\left\{\begin{matrix} w-\frac{c_{0}+c_{1}w+c_{2}w^{2}}{1+d_{1}w+d_{2}w^{2}+d_{3}w^{3}} & P\leq0.05 \\ -\left(w-\frac{c_{0}+c_{1}w+c_{2}w^{2}}{1+d_{1}w+d_{2}w^{2}+d_{3}w^{3}}\right) & P>0.05 \end{matrix}\right.$$

$$w=\sqrt{-2\ln\left(p\right)}$$

where the constant coefficients are as follows: $c_{0}$= 2.515517, $c_{1}$= 0.802853, $c_{2}$= 0.010328, $d_{1}$= 1.432788, $d_{2}$= 0.189269, and $d_{3}$= 0.001308.

**Hierarchical Bayesian Spatiotemporal Model**

In the hierarchical Bayesian spatiotemporal model, all unknown parameters are treated as random variables and accurately described using prior distributions. By introducing multiple levels of random effects, the model can more effectively capture and express the spatiotemporal variation characteristics of disease incidence. This design enables the hierarchical Bayesian spatiotemporal model to exhibit greater flexibility and accuracy in handling complex spatiotemporal data, providing powerful support for research in areas such as disease prediction and environmental monitoring^2^. Given the apparent overdispersion of SFTS case counts, this study assumes that the number of SFTS cases follows a negative binomial distribution:

$$y_{st}\left|\mu_{st},\kappa\sim\right.NegBin\left(\mu_{st},\kappa\right)$$

$$\log\left(\mu_{st}\right)=\log\left(p_{sa\left(t\right)}\right)+\log\left(\rho_{st}\right)$$

where $y_{st}$ represents the number of SFTS cases in county $s$ in month $t$, while $\mu_{st}$ represents the corresponding mean of the distribution, which is equal to the annual average population $\log\left(p_{sa(t}\right)$ multiplied by the unknown estimated SFTS incidence rate $\rho_{st}$ in county $s$ during month $t$. Additionally, $\kappa$ serves as the scale parameter or overdispersion parameter.

We initially constructed a baseline model that incorporates spatiotemporal random effects:

where $\alpha$ represents the intercept, and $\beta_{s\left(s\right)m\left(t\right)}$ represents the monthly random effect at the prefecture level, explaining the seasonality and seasonal autocorrelation of SFTS incidence. By employing a circular first-order random walk prior, we allow the monthly SFTS incidence rate to depend on the previous month, maintaining periodicity without discontinuity between January of $a\left(t\right)$ year and December of year $a\left(t-1\right)$. Additionally, and $\nu_{sy\left(t\right)}$ represent the unstructured and structured spatial random effects at the county level, respectively. Their prior distributions are specified using a modified Besag-York-Mollie (BYM) model. The unstructured spatial random effect accounts for independent confounding factors among study units, such as differences in vector ecology, healthcare access, and reporting rates. The structured spatial random effect, on the other hand, captures common or related environmental and socioeconomic confounding factors (e.g., climate zones, land use) among neighboring study units.

**Distributed Lag Non-linear Model**

In quantitative studies of environmental-health relationships, two key aspects are typically considered: the exposure-response relationship and the lag-response relationship, which refers to the delayed effect of environmental exposure on health risks. The distributed lag non-linear model (DLNM) employs a cross-basis function to simultaneously fit both the nonlinear exposure and its lagged effects, known as the exposure-lag-response relationship^3^. The formula is as follows:

where $f\left(x\right)$ represents the basis function for fitting the exposure-response effect, represents the basis function for fitting the lag-response effect. By combining these two functions, a two-dimensional function is obtained, which is defined as the exposure-lag-response function. This function enables the modeling of both the exposure-response curve along the $x$ direction and the lag-response curve along the direction simultaneously.$x$ represents the observed value of exposure time $t$, while and $L$ correspond to the minimum and maximum lag times, respectively. To appropriately capture the relationship between the independent and dependent variables as well as the distribution of lag effects, suitable basis functions are selected for each component. The tensor product of these two functions results in a cross-basis function, with $\eta$ representing its corresponding parameters^4^.**Modeling Process**

Firstly, the cross-basis functions of four SPEI variables were individually included in the Baseline model to construct the Baseline-SPEI-TEMP-PREC model (Model I):

where $H$ represents the SPEI variable, and represents the cross-basis function of the SPEI indicator. After running the four Baseline-SPEI models separately, the model with the lowest deviance information criterion (DIC) is selected as the optimal Baseline-SPEI model.

Based on the optimal Baseline-SPEI model, the cross-basis functions of three temperature variables were individually incorporated to construct the Baseline-SPEI-TEM model (Model II):

where $T$ denotes the temperature variable, and represents the cross-basis function of temperature. Based on the DIC of each model, the model with the minimum DIC is selected as the Baseline-SPEI-TEM model. Subsequently, the cross-basis function of precipitation is added to the Model II obtained in the previous step, resulting in the final Baseline-SPEI-TEMP-PREC model (Model III):

where $P$ represents the temperature variable, and represents the cross-basis function of temperature. When constructing the model, we set the lag time of the cross-basis functions for the three variables of SPEI, temperature, and precipitation to range from 0 to 6 months. To accurately capture changes in the exposure dimension, we selected a natural cubic spline with two equally spaced internal knots as the basis function. For the lag dimension, we employed a natural cubic spline with one equally spaced internal knot to better reflect the influence of time lag effects on the variables.

To analyze output from the final model, posterior predictive distributions of the response variable were simulated using samples from the posterior distribution of the parameters and hyperparameters from the model, fitted 12 × 12 times, leaving out a month per year each time. The posterior predictive distribution of SFTS cases $y_{st}$ was estimated by drawing 1000 random values from a negative binomial distribution with mean corresponding to the elements of $\mu_{\text{st}}$ and scale parameter corresponding to the elements of the overdispersion parameter $\kappa$ estimated from the model for the prediction month of interest. This step allows for uncertainty in the response variable, given the model parameters. We therefore generated posterior predictive distributions for each timestep across all counties and compared posterior predictive summaries to observed cases.

**Supplementary References**

1. CW Thornthwaite. An approach toward a rational classification of climate. Geographical Review. 1948;38(1):55-94.

2. PL McDermott, CK Wikle, J Millspaugh. A hierarchical spatiotemporal analog forecasting model for count data. Ecology and evolution. 2018;8(1):790-800.

3. A Gasparrini. Distributed Lag Linear and Non-Linear Models in R: The Package dlnm. Journal of statistical software. 2011;43(8):1-20.

4. A Gasparrini, B Armstrong, MG Kenward. Distributed lag non-linear models. Statistics in medicine. 2010;29(21):2224-34.

**Table S1. Explanation and source of variables used in this study.**

| **Variable** | **Explanation of variable (unit)** | **Source of variable** |
| --- | --- | --- |
| Mean temperature | Monthly average temperature (°C) | China Meteorological Data Service Center |
| Maximum temperature | Monthly maximum temperature (°C) |
| Minimum temperature | Monthly minimum temperature (°C) |
| Precipitation | Monthly average precipitation (mm) |
| SPEI-1 | 1-month scale monthly SPEI index | Calculated based on average temperature, precipitation, and latitude |
| SPEI-3 | 3-month scale monthly SPEI index |
| SPEI-6 | 6-month scale monthly SPEI index |
| SPEI-12 | 12-month scale monthly SPEI index |
| Population count | Permanent population count | Statistical yearbooks of counties |
| GDP per capita | gross domestic product per capita (thousand yuan) |
| Proportion of value-added of primary industry | Proportion of value-added of primary industry (including agriculture, forestry, animal husbandry, and fisheries sectors) in total GDP (%) |
| Number of medical institutions per capita | Number of general hospitals, specialty hospitals, community health centers, clinics, and emergency medical centers per 100,000 people |
| Proportion of urban population | Proportion of the permanent population residing in urban areas (%) |
| Proportion of urban construction land | Coverage rate of built-up land in large, medium, and small cities, as well as county-level towns and above (%) | Institute of Geographic Sciences and Natural Resources Research, Chinese Academy of Sciences |

SPEI, Standardized Precipitation Evapotranspiration Index.

**Table S2. Classification of weather conditions based on SPEI.**

| **Types** | **SPEI** |
| --- | --- |
| Extreme wet | SPEI≥2.0 |
| Severe wet | 1.5≤SPEI<2 |
| Moderate wet | 1≤SPEI<1.5 |
| Mild wet | 0.5≤SPEI<1.0 |
| Normal condition | -0.5<SPEI≤0.5 |
| Mild drought | -1.0<SPEI≤-0.5 |
| Moderate drought | -1.5<SPEI≤-1.0 |
| Severe drought | -2.0<SPEI≤-1.5 |
| Extreme drought | SPEI≤−2.0 |

SPEI, Standardized Precipitation Evapotranspiration Index.

The classification of weather conditions was based on the national standards of the People’s Republic of China “Grades of Meteorological Drought (GB/T 20481-2017)”.

**Table S3. The performance of the fitted model in the four-step modeling approach.**

| **Model** | **DIC** |
| --- | --- |
| Baseline model | 44119 |
| Baseline-SPEI model (Baseline + hydrological indicators) |  |
| Baseline model + SPEI-1 | 41793 |
| Baseline model + SPEI-3 | 41891 |
| Baseline model + SPEI-6 | 41856 |
| Baseline model + SPEI-12 | 42795 |
| Baseline-SPEI-TEM model (Baseline + SPEI-1 + temperature indicators) |  |
| Baseline model + SPEI-1 + minimum temperature | 41329 |
| Baseline model + SPEI-1 + maximum temperature | 41325 |
| Baseline model + SPEI-1 + mean temperature | 41410 |
| Baseline-SPEI-TEMP-PREC model (Baseline + SPEI-1+ maximum temperature + precipitation indicators) | |
| Baseline model + SPEI-1 + maximum temperature + precipitation | 41059 |

Baseline model indicates that the model incorporated province-level monthly autocorrelated random effects and annual county-level spatial random effects.

SFTS, severe fever with thrombocytopenia syndrome; DIC, deviance information criterion; SPEI, Standardized Precipitation Evapotranspiration Index.

**Table S4. The estimated numbers of SFTS case by province, and the differences in predicted number of cases compared with the reported number of cases from 2011-2022.**

| **Province** | **2011** | **2012** | **2013** | **2014** | **2015** | **2016** | **2017** | **2018** | **2019** | **2020** | **2021** | **2022** |
| --- | --- | --- | --- | --- | --- | --- | --- | --- | --- | --- | --- | --- |
| Liaoning | 57 (31.58) | 50 (42) | 79 (25.32) | 64 (18.75) | 63 (23.81) | 107 (14.95) | 139 (13.67) | 143 (13.99) | 165 (10.91) | 179 (10.61) | 158 (5.7) | 192 (5.73) |
| Anhui | 86 (29.07) | 86 (31.4) | 107 (24.3) | 259 (11.2) | 293 (9.9) | 499 (5.01) | 357 (6.72) | 345 (4.35) | 492 (9.96) | 674 (4.9) | 572 (4.9) | 885 (5.08) |
| Shandong | 209 (19.62) | 169 (21.3) | 333 (11.11) | 505 (13.86) | 529 (5.67) | 654 (3.82) | 559 (8.41) | 747 (1.34) | 550 (5.09) | 719 (1.53) | 1004 (-0.2) | 1046 (3.63) |
| Henan | 158 (12.66) | 293 (-7.51) | 258 (5.81) | 505 (11.09) | 1019 (3.34) | 883 (0.45) | 528 (-3.41) | 278 (2.16) | 351 (11.97) | 534 (10.67) | 393 (2.04) | 535 (9.53) |
| Hubei | 139 (23.74) | 129 (17.83) | 171 (13.45) | 152 (14.47) | 207 (9.66) | 395 (1.77) | 281 (7.12) | 259 (8.49) | 326 (10.43) | 347 (20.17) | 359 (7.24) | 661 (9.53) |

The number in parentheses was derived from the following formula: $\frac{predicted number of cases - reported number of cases}{reported number of cases}*100\%$*.*

SFTS, severe fever with thrombocytopenia syndrome.

**Table S5. Maximum and minimum relative risk of SFTS associated with maximum temperature for each month within 0-6 months.**

| Lag (month) | Maximum *RR* | |  | Minimum *RR* | |
| --- | --- | --- | --- | --- | --- |
|  | Temperature (℃) | *RR* (95%CI) |  | Temperature (℃) | *RR* (95%CI) |
| 0 | 25.67 | 1.38 (1.26-1.51) |  | 2.57 | 0.18 (0.15-0.23) |
| 1 | 24.89 | 1.19 (1.14-1.23) |  | 2.21 | 0.34 (0.30-0.39) |
| 2 | 23.02 | 1.05 (1.03-1.07) |  | 1.08 | 0.58 (0.52-0.65) |
| 3 | 18.35 | 1.00 (0.99-1.01) |  | 35 | 0.72 (0.62-0.83) |
| 4 | 11.5 | 1.05 (0.99-1.11) |  | 35 | 0.63 (0.56-0.72) |
| 5 | 12.65 | 1.04 (0.99-1.10) |  | 35 | 0.56 (0.46-0.67) |
| 6 | 16.13 | 1.02 (0.98-1.06) |  | 35 | 0.48 (0.34-0.68) |

SFTS, severe fever with thrombocytopenia syndrome; RR, relative risk; CI, confidence interval.

The reference group was the median monthly maximum temperature (19 °C).

**Table S6. Maximum and minimum relative risk of SFTS associated with precipitation for each month within 0-6 months.**

| Lag (month) | Maximum *RR* | |  | Minimum *RR* | |
| --- | --- | --- | --- | --- | --- |
|  | Precipitation (mm) | *RR* (95%CI) |  | Precipitation (mm) | *RR* (95%CI) |
| 0 | 200 | 1.48 (1.30-1.69) |  | 0 | 0.45 (0.39-0.52) |
| 1 | 200 | 1.57 (1.44-1.71) |  | 0 | 0.45 (0.41-0.50) |
| 2 | 200 | 1.64 (1.50-1.79) |  | 0 | 0.47 (0.42-0.52) |
| 3 | 200 | 1.65 (1.50-1.82) |  | 0 | 0.49 (0.43-0.55) |
| 4 | 200 | 1.59 (1.45-1.74) |  | 0 | 0.52 (0.47-0.58) |
| 5 | 200 | 1.48 (1.33-1.64) |  | 0 | 0.57 (0.52-0.63) |
| 6 | 200 | 1.37 (1.20-1.56) |  | 0 | 0.62 (0.54-0.73) |

SFTS, severe fever with thrombocytopenia syndrome; RR, relative risk; CI, confidence interval.

The reference group was the median monthly precipitation (70 mm).

**Table S7. Maximum and minimum relative risk of SFTS associated with drought conditions (SPEI≤-0.5) for each month within 0-6 months.**

| Lag (month) | Maximum *RR* | |  | Minimum *RR* | |
| --- | --- | --- | --- | --- | --- |
|  | SPEI | *RR* (95%CI) |  | SPEI | *RR* (95%CI) |
| 0 | -2.5 | 1.21 (1.04-1.40) |  | -0.90 | 0.95 (0.92-0.98) |
| 1 | -2.5 | 1.22 (1.09-1.36) |  | -0.81 | 0.97 (0.95-0.99) |
| 2 | -2.5 | 1.22 (1.08-1.37) |  | -0.69 | 0.98 (0.96-0.9993) |
| 3 | -2.5 | 1.21 (1.06-1.37) |  | -0.54 | 0.99 (0.98-1.01) |
| 4 | -2.5 | 1.18 (1.04-1.33) |  | -0.50 | 0.997 (0.98-1.01) |
| 5 | -2.5 | 1.13 (0.98-1.31) |  | -0.50 | 1.00 (0.99-1.02) |
| 6 | -2.5 | 1.09 (0.88-1.35) |  | -0.50 | 1.01 (0.99-1.03) |

SFTS, severe fever with thrombocytopenia syndrome; SPEI, Standardized Precipitation Evapotranspiration Index; RR, relative risk; CI, confidence interval.

The reference group was an SPEI value of 0 for normal hydrological conditions.

**Table S8. Maximum and minimum relative risk of SFTS associated with wet conditions (SPEI≥0.5) for each month within 0-6 months.**

| Lag (month) | Maximum *RR* | |  | Minimum *RR* | |
| --- | --- | --- | --- | --- | --- |
|  | SPEI | *RR* (95%CI) |  | SPEI | *RR* (95%CI) |
| 0 | 1.31 | 1.07 (1.03-1.11) |  | 0.50 | 1.04 (1.02-1.06) |
| 1 | 1.81 | 1.07 (1.02-1.13) |  | 0.50 | 1.03 (1.02-1.05) |
| 2 | 2.5 | 1.09 (0.99-1.20) |  | 0.50 | 1.03 (1.01-1.04) |
| 3 | 2.5 | 1.10 (0.99-1.21) |  | 0.50 | 1.02 (1.00-1.03) |
| 4 | 2.5 | 1.08 (0.99-1.19) |  | 0.50 | 1.01 (1.00-1.03) |
| 5 | 2.5 | 1.06 (0.96-1.16) |  | 0.50 | 1.00 (1.00-1.02) |
| 6 | 2.5 | 1.03 (0.90-1.17) |  | 0.5 | 0.997 (0.98-1.01) |

SFTS, severe fever with thrombocytopenia syndrome; SPEI, Standardized Precipitation Evapotranspiration Index; RR, relative risk; CI, confidence interval.

The reference group was an SPEI value of 0 for normal hydrological conditions.

**Table S9.** **The performance of the model fit in the four-step modeling approach on four geographical clusters with SFTS.**

| **Model** | **DIC** |
| --- | --- |
| **Cluster I** |  |
| Baseline model | 3626 |
| Baseline-SPEI model (Baseline + hydrological indicators) |  |
| Baseline model + SPEI-1 | 3155 |
| Baseline model + SPEI-3 | 3151 |
| Baseline model + SPEI-6 | 3166 |
| Baseline model + SPEI-12 | 3165 |
| Baseline-SPEI-TEM model (Baseline + SPEI-3+ temperature indicators) |  |
| Baseline model + SPEI-3 + minimum temperature | 3119 |
| Baseline model + SPEI-3 + maximum temperature | 3127 |
| Baseline model + SPEI-3 + mean temperature | 3124 |
| Baseline-SPEI-TEMP-PREC model (Baseline + SPEI-3+ minimum temperature + precipitation indicators) | |
| Baseline model + SPEI-3 + minimum temperature + precipitation | 3114 |
| **Cluster II** |  |
| Baseline model | 5270 |
| Baseline-SPEI model (Baseline + hydrological indicators) |  |
| Baseline model + SPEI-1 | 5273 |
| Baseline model + SPEI-3 | 5275 |
| Baseline model + SPEI-6 | 5274 |
| Baseline model + SPEI-12 | 5257 |
| Baseline-SPEI-TEM model (Baseline + SPEI-12+ temperature indicators) |  |
| Baseline model + SPEI-12 + minimum temperature | 5257 |
| Baseline model + SPEI-12 + maximum temperature | 5242 |
| Baseline model + SPEI-12 + mean temperature | 5247 |
| Baseline-SPEI-TEMP-PREC model (Baseline + SPEI-12+ maximum temperature + precipitation indicators) | |
| Baseline model + SPEI-12 + maximum temperature + precipitation | 5238 |
| **Cluster III** |  |
| Baseline model | 11006 |
| Baseline-SPEI model (Baseline + hydrological indicators) |  |
| Baseline model + SPEI-1 | 8819 |
| Baseline model + SPEI-3 | 8826 |
| Baseline model + SPEI-6 | 8832 |
| Baseline model + SPEI-12 | 8813 |
| Baseline-SPEI-TEM model (Baseline + SPEI-12+ temperature indicators) |  |
| Baseline model + SPEI-12 + minimum temperature | 8781 |
| Baseline model + SPEI-12 + maximum temperature | 8782 |
| Baseline model + SPEI-12 + mean temperature | 8780 |
| Baseline-SPEI-TEMP-PREC model (Baseline + SPEI-12+ mean temperature + precipitation indicators) | |
| Baseline model + SPEI-12 + mean temperature + precipitation | 8763 |
| **Cluster IV** |  |
| Baseline model | 30003 |
| Baseline-SPEI model (Baseline + hydrological model) |  |
| Baseline model + SPEI-1 | 29680 |
| Baseline model + SPEI-3 | 29739 |
| Baseline model + SPEI-6 | 29868 |
| Baseline model + SPEI-12 | 29371 |
| Baseline-SPEI-TEM model (Baseline + SPEI-12+ temperature indicators) |  |
| Baseline model + SPEI-12 + minimum temperature | 29280 |
| Baseline model + SPEI-12 + maximum temperature | 29956 |
| Baseline model + SPEI-12 + mean temperature | 29436 |
| Baseline-SPEI-TEMP-PREC model (Baseline + SPEI-12+ minimum temperature + precipitation indicators) | |
| Baseline model + SPEI-12 + minimum temperature + precipitation | 27588 |

The spatial distribution of four geographic clusters of SFTS cases was shown in Figure S1.

Baseline model indicates that the model incorporated province-level monthly autocorrelated random effects and annual county-level spatial random effects.

SFTS, severe fever with thrombocytopenia syndrome; DIC, deviance information criterion; SPEI, Standardized Precipitation Evapotranspiration Index.

**Table S10. Maximum relative risk of SFTS for meteorological factor within 6 months.**

| Cluster | Meteorological factor | Maximum *RR* | | |  | Maximum cumulative *RR* | |
| --- | --- | --- | --- | --- | --- | --- | --- |
|  |  | Value | Lag (month) | *RR* (95%CI) |  | Value | *RR* (95%CI) |
| Cluster I | Minimum temperature | 11.38℃ | 0 | 1.35 (1.07‒1.70) |  | 8.61℃ | 1.68 (1.07‒2.63) |
| Cluster I | Precipitation | 120mm | 3 | 1.68 (1.07‒2.63) |  | 120mm | 4.70 (1.53‒14.37) |
| Cluster I | SPEI-3 | -2 | 3 | 1.35 (1.12‒1.63) |  | -2 | 2.04 (0.70‒5.95) |
| Cluster II | Maximum temperature | 30℃ | 1 | 1.74 (1.25‒2.42) |  | 5℃ | 1.22 (0.25‒5.84) |
| Cluster II | Precipitation | 0mm | 6 | 1.15 (0.94‒1.40) |  | 140mm | 1.16 (0.30‒4.53) |
| Cluster II | SPEI-12 | 1.5 | 6 | 1.52 (1.04‒2.21) |  | 1.5 | 5.66 (3.39‒9.45) |
| Cluster III | Mean temperature | 0℃ | 6 | 1.74 (1.24‒2.45) |  | 18.13℃ | 1.59 (1.00‒2.52) |
| Cluster III | Precipitation | 101mm | 3 | 1.11 (1.00‒1.23) |  | 81mm | 1.33 (1.00‒1.78) |
| Cluster III | SPEI-12 | 2 | 6 | 2.34 (1.45‒3.76) |  | 2 | 4.45 (2.73‒7.24) |
| Cluster IV | Minimum temperature | 1.5℃ | 4 | 1.38 (1.25‒1.53) |  | 8.3℃ | 1.09 (0.79‒1.49) |
| Cluster IV | Precipitation | 200mm | 3 | 1.31 (1.18‒1.44) |  | 183mm | 2.67 (1.66‒4.31) |
| Cluster IV | SPEI-12 | 2 | 6 | 1.57 (1.37‒1.79) |  | -2 | 3.41 (2.16‒5.38) |

SFTS, severe fever with thrombocytopenia syndrome; RR, relative risk; CI, confidence interval; SPEI, Standardized Precipitation Evapotranspiration Index.

The spatial distribution of four geographic clusters of SFTS cases was shown in Figure S1.

The reference groups were the median monthly maximum temperature (19 °C), the median monthly precipitation (70 mm), and an SPEI value of 0 for normal hydrological conditions.

**Table S11. The DIC of the model after incorporating the interaction terms between climatic and socioeconomic factors in the final model.**

| Socioeconomic factors | SPEI-1 | Maximum temperature | Precipitation |
| --- | --- | --- | --- |
| GDP per capita | 40715 | 40758 | 40890 |
| proportion of value-added of primary industry | 40895 | 40866 | 41049 |
| numbers of medical institutions per capita | 40618 | 41004 | 40591 |
| proportion of urban construction land | 41050 | 41028 | 40417 |
| proportion of urban population | 40915 | 40959 | 41051 |

DIC, deviance information criterion

**Table S12.** **Maximum and cumulative relative risk of SFTS for meteorological factors within 6 months by different scenarios of proportion of value-added of primary industry.**

| Meteorological factor | Proportion of value-added of primary industry | Maximum *RR* | | |  | Maximum cumulative *RR* | |
| --- | --- | --- | --- | --- | --- | --- | --- |
|  |  | Value | Lag (month) | *RR* (95%CI) |  | Value | *RR* (95%CI) |
| Maximum temperature | High | 26.9℃ | 0 | 1.51 (1.31‒1.73) |  | 23.2℃ | 1.36 (1.10‒1.68) |
|  | Median | 25.2℃ | 0 | 1.32 (1.20‒1.44) |  | 21.4℃ | 1.14 (1.03‒1.27) |
|  | Low | 24.2℃ | 0 | 1.22 (1.09‒1.36) |  | 20.4℃ | 1.09 (1.00‒1.18) |
| Precipitation | High | 200 mm | 3 | 1.64 (1.46‒1.84) |  | 200 mm | 21.75 (11.79‒40.11) |
|  | Median | 200 mm | 2 | 1.66 (1.52‒1.13) |  | 200 mm | 18.11 (10.90‒30.09) |
|  | Low | 200 mm | 2 | 1.74 (1.55‒1.94) |  | 200 mm | 16.60 (8.20‒33.61) |
| SPEI-1 at drought conditions (SPEI-1≤-0.5) | High | -2.5 | 6 | 1.65 (1.19‒2.28) |  | -2.5 | 10.01 (3.88‒25.85) |
|  | Median | -2.5 | 0 | 1.24 (1.06‒1.45) |  | -2.5 | 3.42 (1.72‒6.77) |
|  | Low | -2.5 | 2 | 1.06 (0.88‒1.29) |  | -0.5 | 0.99 (0.86‒1.29) |
| SPEI-1 at wet conditions (SPEI-1≥0.5) | High | 2.1 | 0 | 1.21 (1.00-1.45) |  | 2 | 2.10 (1.03-4.30) |
|  | Median | 1.6 | 0 | 1.10 (1.03-1.16) |  | 2.2 | 1.62 (1.00-2.61) |
|  | Low | 2.5 | 3 | 1.08 (0.92-1.28) |  | 2.5 | 1.17 (0.50-2.76) |

SFTS, severe fever with thrombocytopenia syndrome; RR, relative risk; CI, confidence interval; SPEI, Standardized Precipitation Evapotranspiration Index.

The different levels of proportion of value-added of primary industry were obtained by centralizing each county to the 25th, 50th, and 75th percentiles.

The impacts of SPEI on the risk of SFTS are presented under drought and wet conditions, respectively.

The reference groups were the median monthly maximum temperature (19 °C), the median monthly precipitation (70 mm), and an SPEI value of 0 for normal hydrological conditions.

**Table S13. Maximum and cumulative relative risk of SFTS for meteorological factors within 6 months by different scenarios of numbers of medical institutions per capita.**

| Meteorological factor | Numbers of medical institutions per capita | Maximum *RR* | | |  | Maximum cumulative *RR* | |
| --- | --- | --- | --- | --- | --- | --- | --- |
|  |  | Value | Lag (month) | *RR* (95%CI) |  | Value | *RR* (95%CI) |
| Maximum temperature | High | 23.7℃ | 0 | 1.17 (1.07‒1.28) |  | 20.1℃ | 1.06 (1.00‒1.13) |
|  | Median | 26.2℃ | 0 | 1.45 (1.31‒1.61) |  | 22.3℃ | 1.28 (1.11‒1.47) |
|  | Low | 26.9℃ | 0 | 1.55 (1.36‒1.77) |  | 22.5℃ | 1.35 (1.15‒1.60) |
| Precipitation | High | 200mm | 4 | 1.66 (1.48‒1.85) |  | 200mm | 25.39 (13.55‒47.55) |
|  | Median | 200mm | 2 | 1.70 (1.55‒1.86) |  | 200mm | 20.51 (12.28‒34.25) |
|  | Low | 200mm | 2 | 1.73 (1.56‒1.91) |  | 200mm | 19.82 (11.02‒35.63) |
| SPEI-1 at drought conditions (SPEI-1≤-0.5) | High | -2.5 | 0 | 1.19 (1.01‒1.41) |  | -2.5 | 2.31 (1.04‒5.14) |
|  | Median | -2.5 | 1 | 1.22 (1.09‒1.37) |  | -2.5 | 3.07 (1.52‒6.20) |
|  | Low | -2.5 | 2 | 1.23 (1.06‒1.42) |  | -2.5 | 3.42 (1.56‒7.49) |
| SPEI-1 at wet conditions (SPEI-1≥0.5) | High | 2.5 | 3 | 1.15 (1.03-1.29) |  | 2.5 | 1.94 (1.08-3.50) |
|  | Median | 1.8 | 2 | 1.06 (1.00-1.12) |  | 1.8 | 1.39 (1.02-1.90) |
|  | Low | 1.2 | 0 | 1.05 (1.00-1.10) |  | 1.4 | 1.29 (1.01-1.64) |

SFTS, severe fever with thrombocytopenia syndrome; RR, relative risk; CI, confidence interval; SPEI, Standardized Precipitation Evapotranspiration Index.

The different levels of proportion of numbers of medical institutions per capita were obtained by centralizing each county to the 25th, 50th, and 75th percentiles.

The impacts of SPEI on the risk of SFTS are presented under drought and wet conditions, respectively.

The reference groups were the median monthly maximum temperature (19 °C), the median monthly precipitation (70 mm), and an SPEI value of 0 for normal hydrological conditions.

**Table S14. Maximum and cumulative relative risk of SFTS for meteorological factors within 6 months by different scenarios of proportion of urban construction land.**

| Meteorological factor | Proportion of urban construction land | Maximum *RR* | | |  | Maximum cumulative *RR* | |
| --- | --- | --- | --- | --- | --- | --- | --- |
|  |  | Value | Lag (month) | *RR* (95%CI) |  | Value | *RR* (95%CI) |
| Maximum temperature | High | 23.8℃ | 0 | 1.19 (1.06‒1.34) |  | 20.4℃ | 1.10 (1.00‒1.21) |
|  | Median | 25.6℃ | 0 | 1.33 (1.21‒1.45) |  | 21.7℃ | 1.16 (1.04‒1.30) |
|  | Low | 25.8℃ | 0 | 1.35 (1.22‒1.48) |  | 21.8℃ | 1.18 (1.05‒1.32) |
| Precipitation | High | 200mm | 3 | 1.95 (1.65‒2.30) |  | 200mm | 16.82 (5.76‒49.08) |
|  | Median | 200mm | 3 | 1.64 (1.48‒1.80) |  | 200mm | 18.06 (11.18‒29.19) |
|  | Low | 200mm | 3 | 1.62 (1.46‒1.78) |  | 200mm | 18.39 (11.30‒29.94) |
| SPEI-1 at drought conditions (SPEI-1≤-0.5) | High | -2.5 | 0 | 1.14 (0.79‒1.66) |  | -2.5 | 1.79 (0.44‒7.27) |
|  | Median | -2.5 | 0 | 1.17 (1.00‒1.36) |  | -2.5 | 2.10 (1.04‒4.25) |
|  | Low | -2.5 | 1 | 1.17 (1.05‒1.31) |  | -2.5 | 2.09 (1.02‒4.29) |
| SPEI-1 at wet conditions (SPEI-1≥0.5) | High | -0.7 | 5 | 1.02 (0.97‒1.08) |  | -0.6 | 1.06 (0.80‒1.40) |
|  | Median | -2.5 | 1 | 1.14 (1.02‒1.28) |  | -2.5 | 1.68 (0.80‒3.52) |
|  | Low | -2.5 | 2 | 1.39 (1.20‒1.61) |  | -2.5 | 5.53 (2.57‒11.89) |

SFTS, severe fever with thrombocytopenia syndrome; RR, relative risk; CI, confidence interval; SPEI, Standardized Precipitation Evapotranspiration Index.

The different levels of proportion of urban construction land were obtained by centralizing each county to the 25th, 50th, and 75th percentiles.

The impacts of SPEI on the risk of SFTS are presented under drought and wet conditions, respectively.

The reference groups were the median monthly maximum temperature (19 °C), the median monthly precipitation (70 mm), and an SPEI value of 0 for normal hydrological conditions.

**Table S15. Maximum and cumulative relative risk of SFTS for meteorological factors within 6 months by different scenarios of proportion of urban population.**

| Meteorological factor | Proportion of urban population | Maximum *RR* | | |  | Maximum cumulative *RR* | |
| --- | --- | --- | --- | --- | --- | --- | --- |
|  |  | Value | Lag (month) | *RR* (95%CI) |  | Value | *RR* (95%CI) |
| Maximum temperature | High | 25.6℃ | 0 | 1.28 (1.09‒1.51) |  | 19.3℃ | 1.02 (1.00‒1.04) |
|  | Median | 25.6℃ | 0 | 1.33 (1.21‒1.46) |  | 21.3℃ | 1.12 (1.01‒1.23) |
|  | Low | 25.5℃ | 0 | 1.33 (1.19‒1.48) |  | 21.4℃ | 1.12 (1.00‒1.26) |
| Precipitation | High | 200mm | 2 | 1.73 (1.50‒2.00) |  | 200mm | 11.52 (4.47‒29.71) |
|  | Median | 200mm | 3 | 1.67 (1.52‒1.85) |  | 200mm | 20.45 (12.63‒33.11) |
|  | Low | 200mm | 3 | 1.68 (1.51‒1.87) |  | 200mm | 26.10 (15.01‒45.39) |
| SPEI-1 at drought conditions (SPEI-1≤-0.5) | High | -0.7 | 5 | 1.02 (0.97‒1.08) |  | -0.6 | 1.06 (0.80‒1.40) |
|  | Median | -2.5 | 1 | 1.14 (1.02‒1.28) |  | -2.5 | 1.68 (0.80‒3.52) |
|  | Low | -2.5 | 2 | 1.39 (1.20‒1.61) |  | -2.5 | 5.53 (2.57‒11.89) |
| SPEI-1 at wet conditions (SPEI-1≥0.5) | High | 2.5 | 0 | 1.44 (1.00-2.06) |  | 2.5 | 5.27 (1.12-24.74) |
|  | Median | 2.5 | 3 | 1.12 (1.01-1.24) |  | 2.5 | 1.71 (1.00-2.92) |
|  | Low | 1 | 1 | 1.05 (1.02-1.08) |  | 1.1 | 1.28 (1.06-1.53) |

SFTS, severe fever with thrombocytopenia syndrome; RR, relative risk; CI, confidence interval; SPEI, Standardized Precipitation Evapotranspiration Index.

The different levels of proportion of urban population were obtained by centralizing each county to the 25th, 50th, and 75th percentiles.

The impacts of SPEI on the risk of SFTS are presented under drought and wet conditions, respectively.

The reference groups were the median monthly maximum temperature (19 °C), the median monthly precipitation (70 mm), and an SPEI value of 0 for normal hydrological conditions.

**
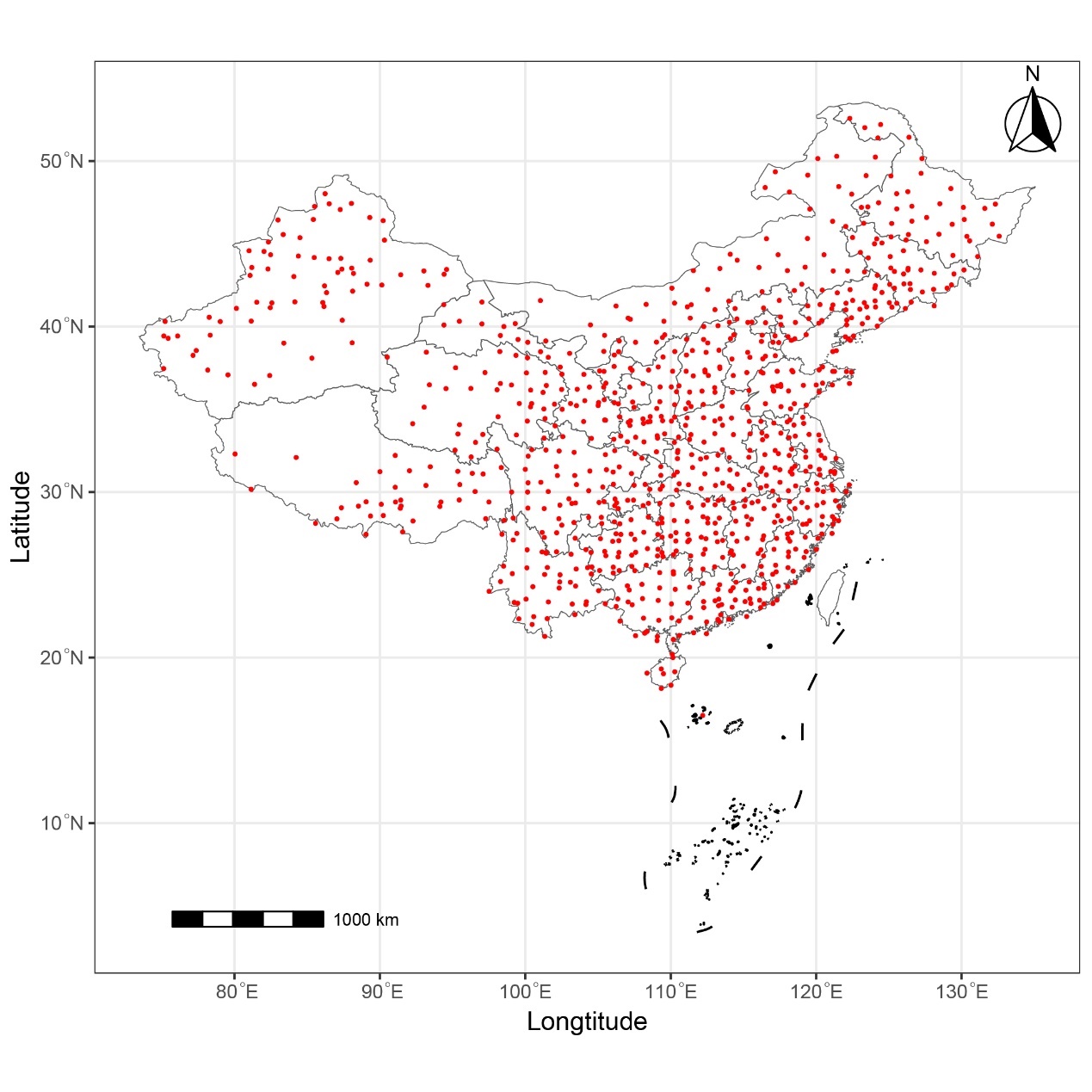
**

**Figure S1. Geographical distribution of 890 weather surveillance stations in China.**


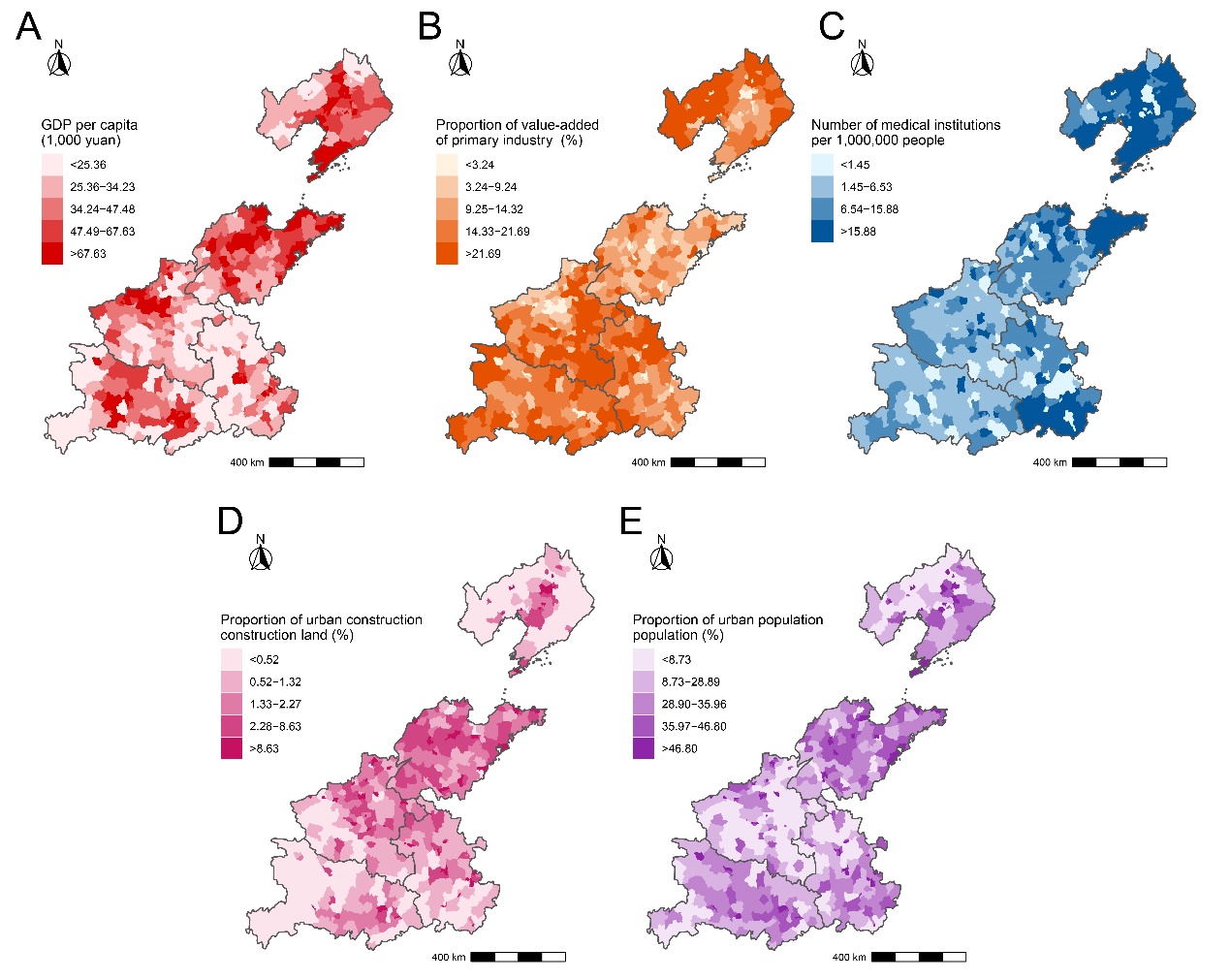


**Figure S2. Spatial distribution of annual mean value of socioeconomic factors for the 604 counties of the five high-incidence provinces in China from 2011-2022.**

(A) GDP per capita. (B) Proportion of value-added of primary industry. (C) Number of medical institutions per capita. (D) Proportion of urban construction land. (E) Proportion of urban population


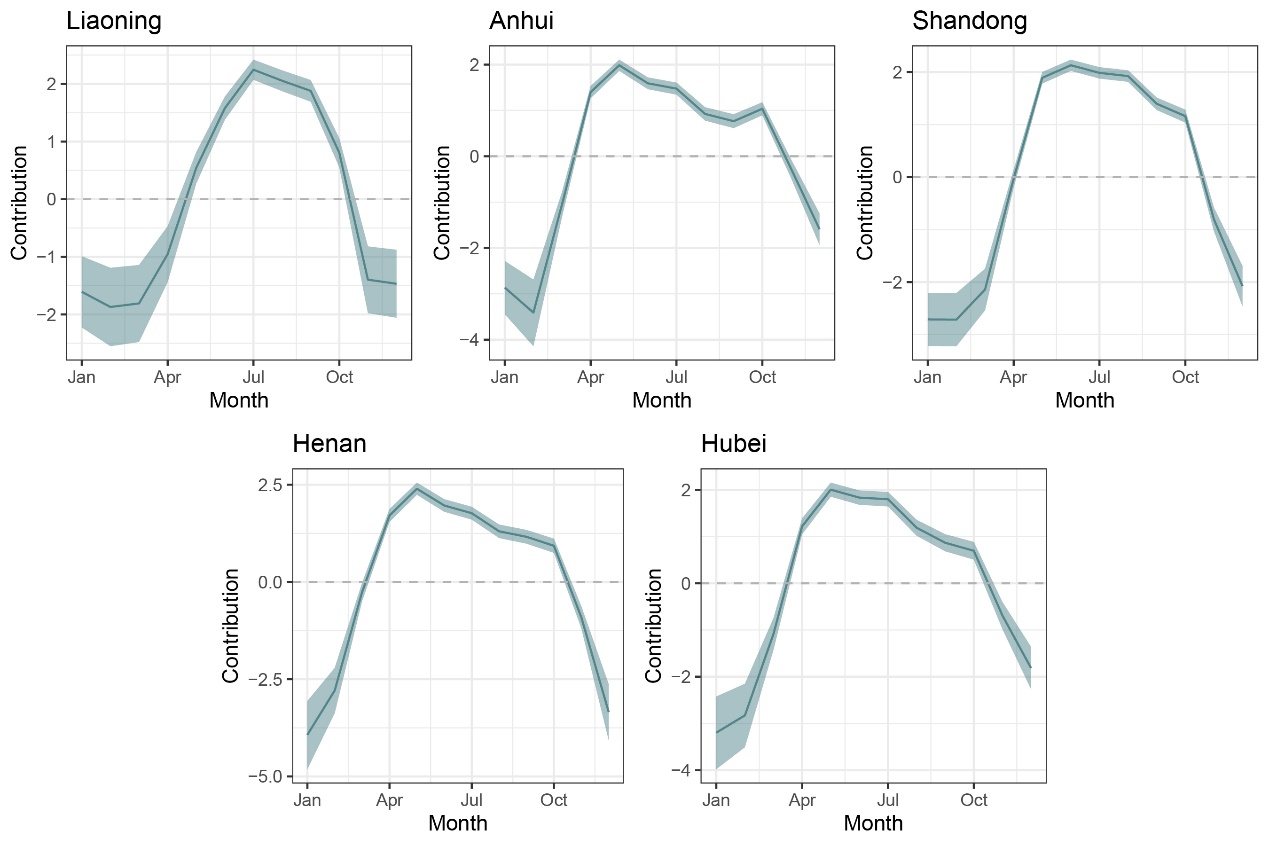


**Figure S3. Posterior distributions of province-specific autocorrelated monthly random effects.**

Posterior mean (solid curve) and 95% credible interval (shaded area) of the marginal posterior distribution of the autocorrelated month random effects at the linear predictor scale from January to December for the five provinces.


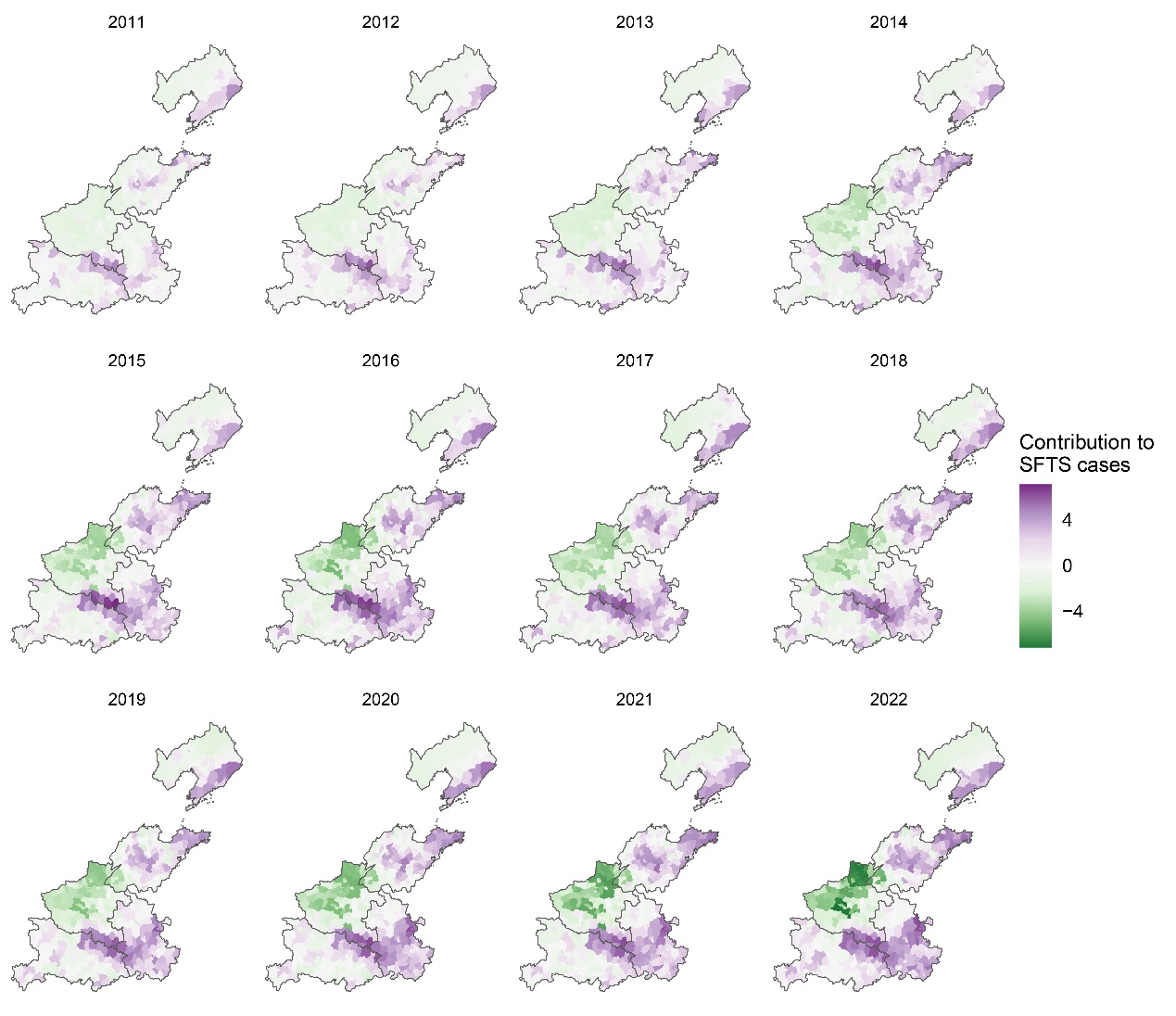


**Figure S4. Contribution of year-specific spatial random effects to the number of SFTS cases estimates.**

Marginal posterior mean of the combined spatially structured and unstructured random effects at the linear predictor scale per year from 2011 to 2022.


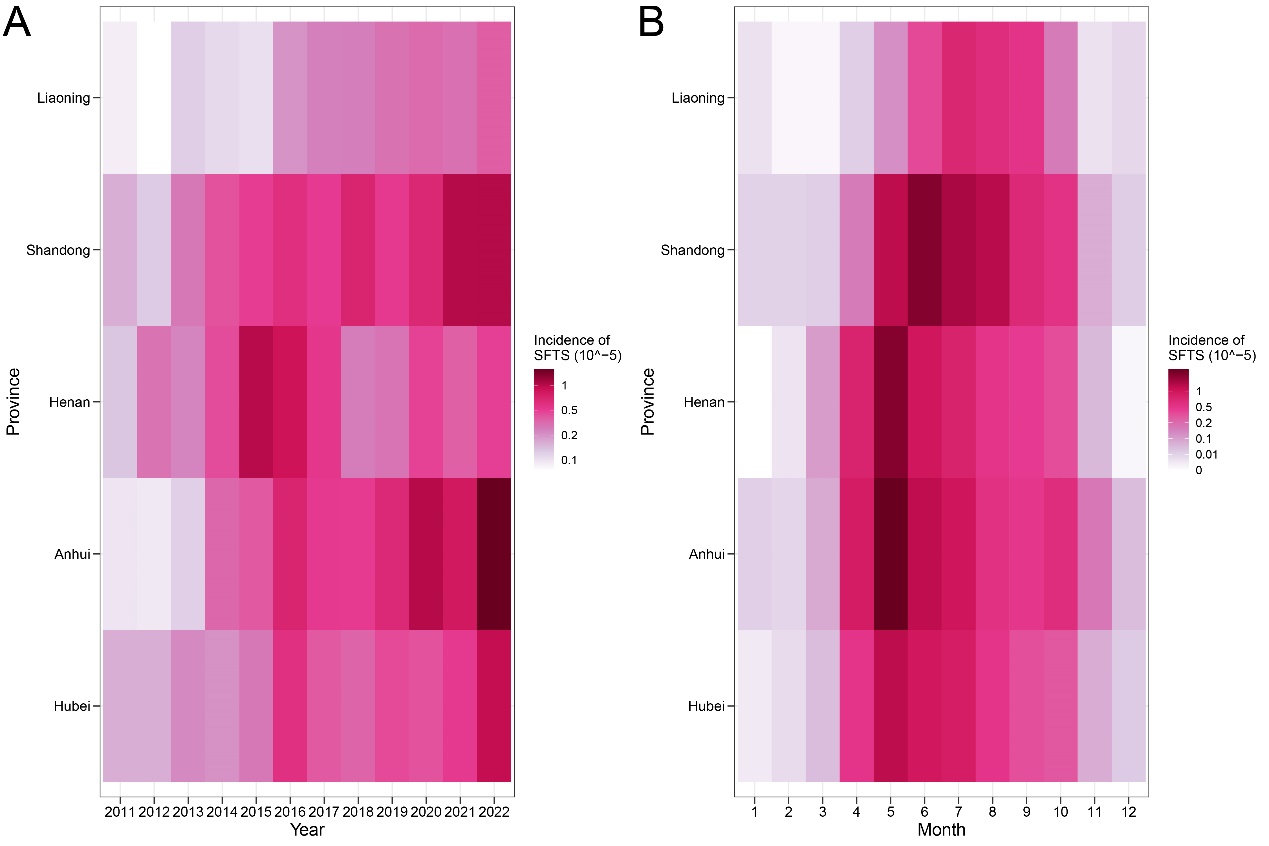


**Figure S5. The annual and monthly incidence rates of SFTS of the five high-incidence provinces** **from 2011 to 2022.**

(A) Annual incidence rates of SFTS. (B) Monthly incidence rates of SFTS. SFTS, severe fever with thrombocytopenia syndrome.


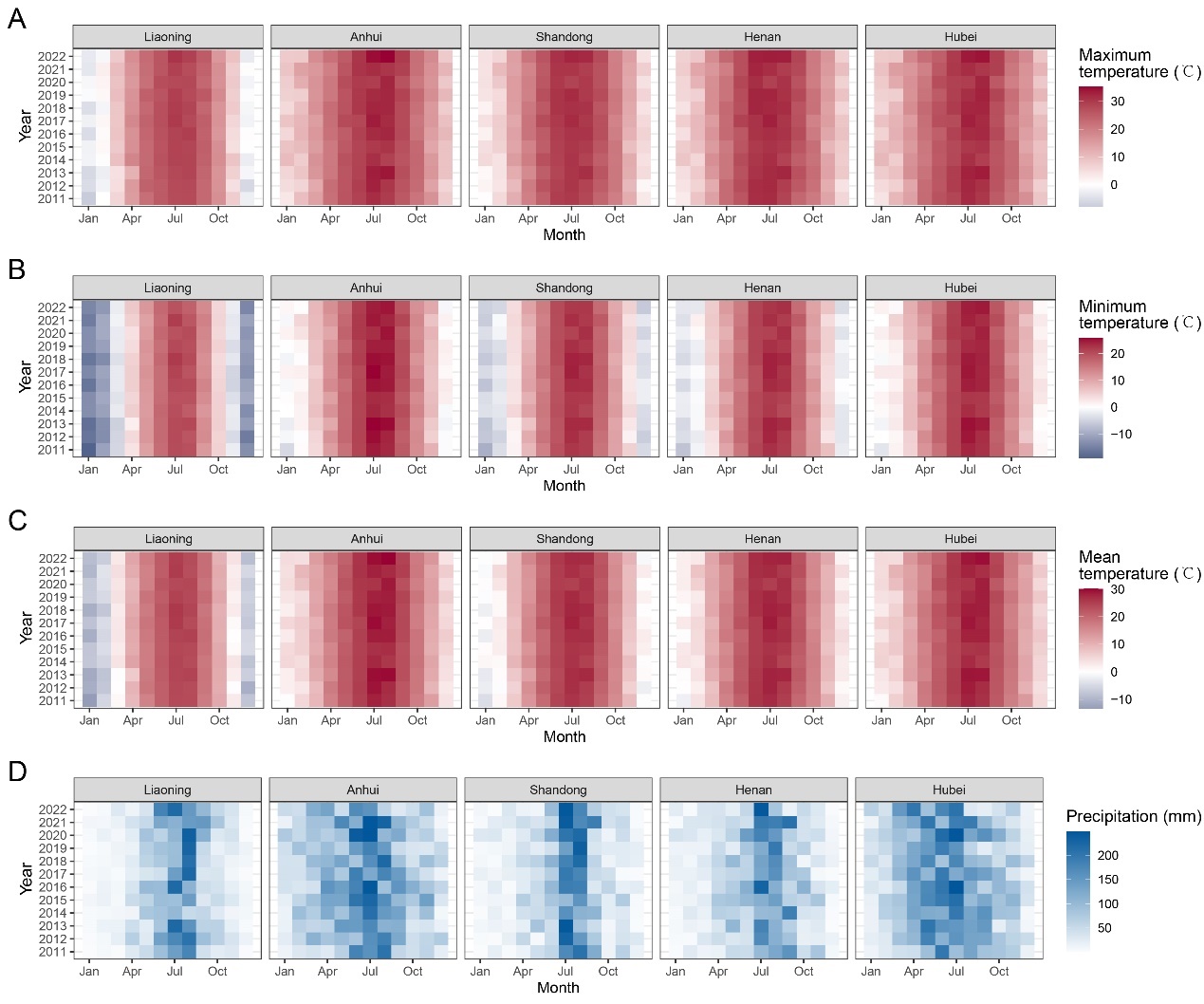


**Figure S6. Monthly maximum temperature, minimum temperature, mean temperature, and precipitation of the five high-incidence provinces from 2011 to 2022.**

(A) Monthly maximum temperature. (B) Monthly minimum temperature. (C) Monthly mean temperature. (D) Monthly precipitation.


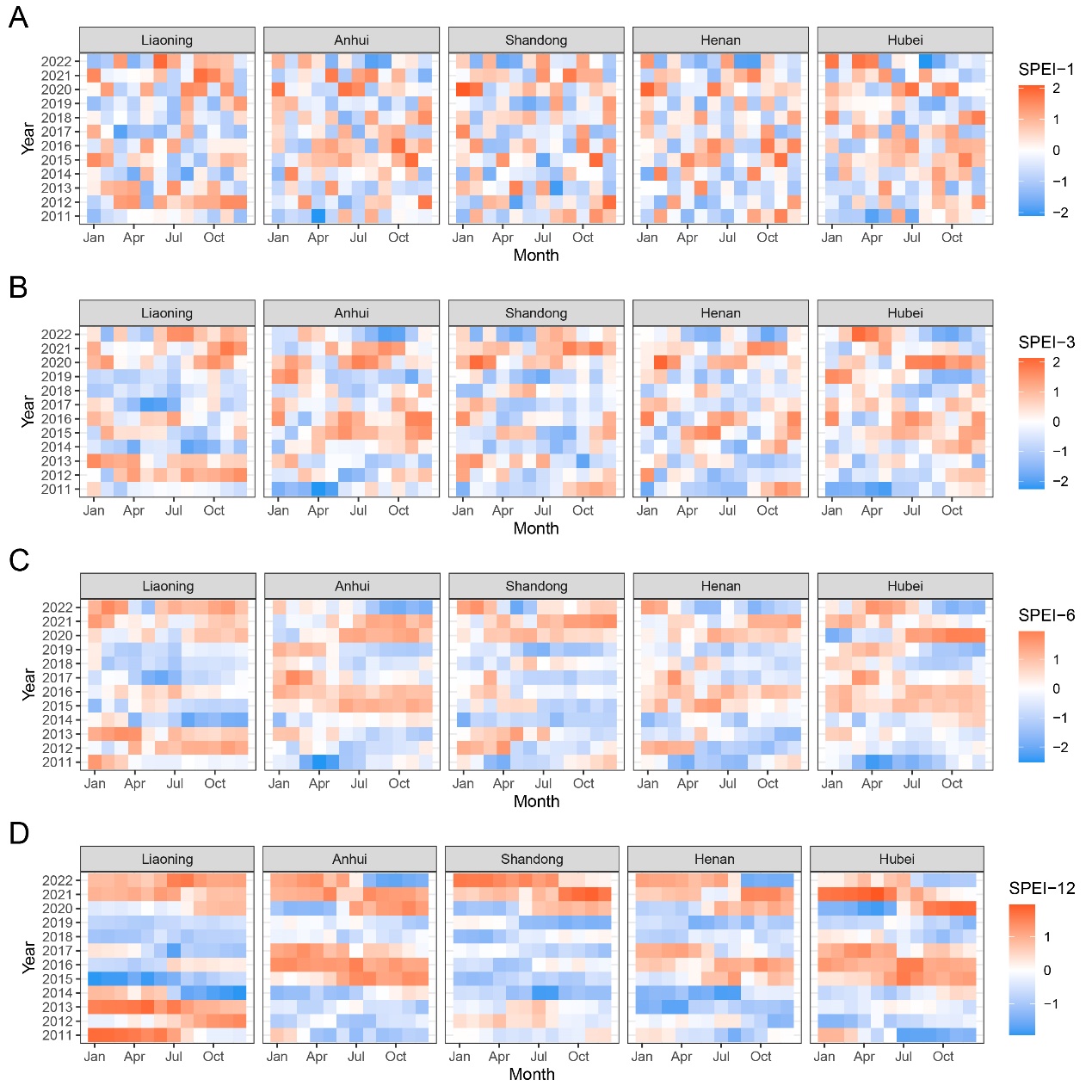


**Figure S7. Monthly SPEI-1, SPEI-3, SPEI-6, and SPEI-12 of the five high-incidence provinces from 2011 to 2022.**

(A) Monthly SPEI-1. (B) Monthly SPEI-3. (C) Monthly SPEI-6. (D) Monthly SPEI-12.

SPEI, Standardized Precipitation Evapotranspiration Index.


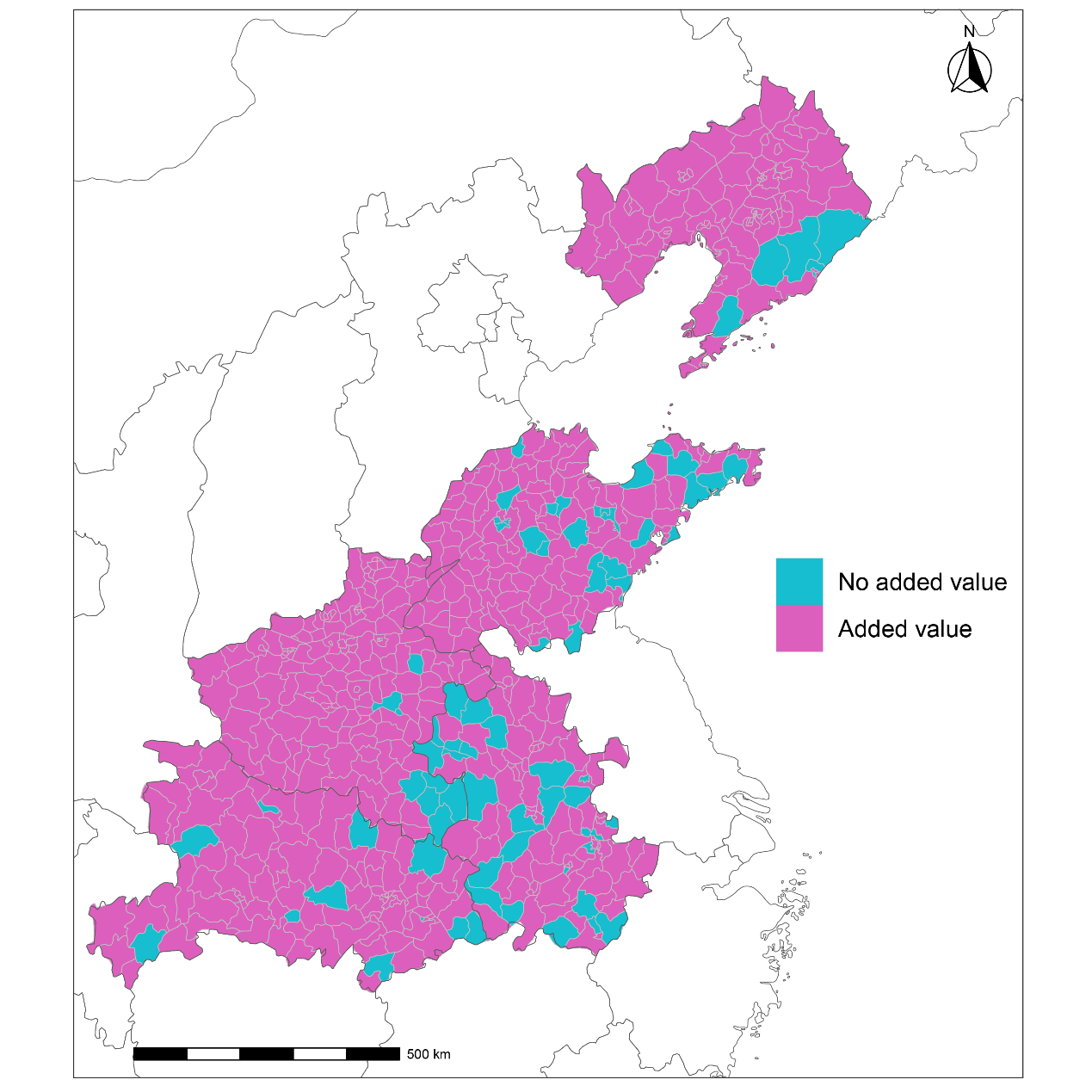


**Figure S8. Added value of using final model compared to the baseline model.**

Difference between mean absolute error (MAE) for the baseline model and MAE for the final model. Counties with positive values (pink) suggest that capturing the nonlinear and delayed impacts of temperature, precipitation, and SPEI-1 improves the model in these areas. Counties with negative values (blue) suggest that climate information did not improve the model fit and other unexplained factors may dominate space-time dynamics in these areas.


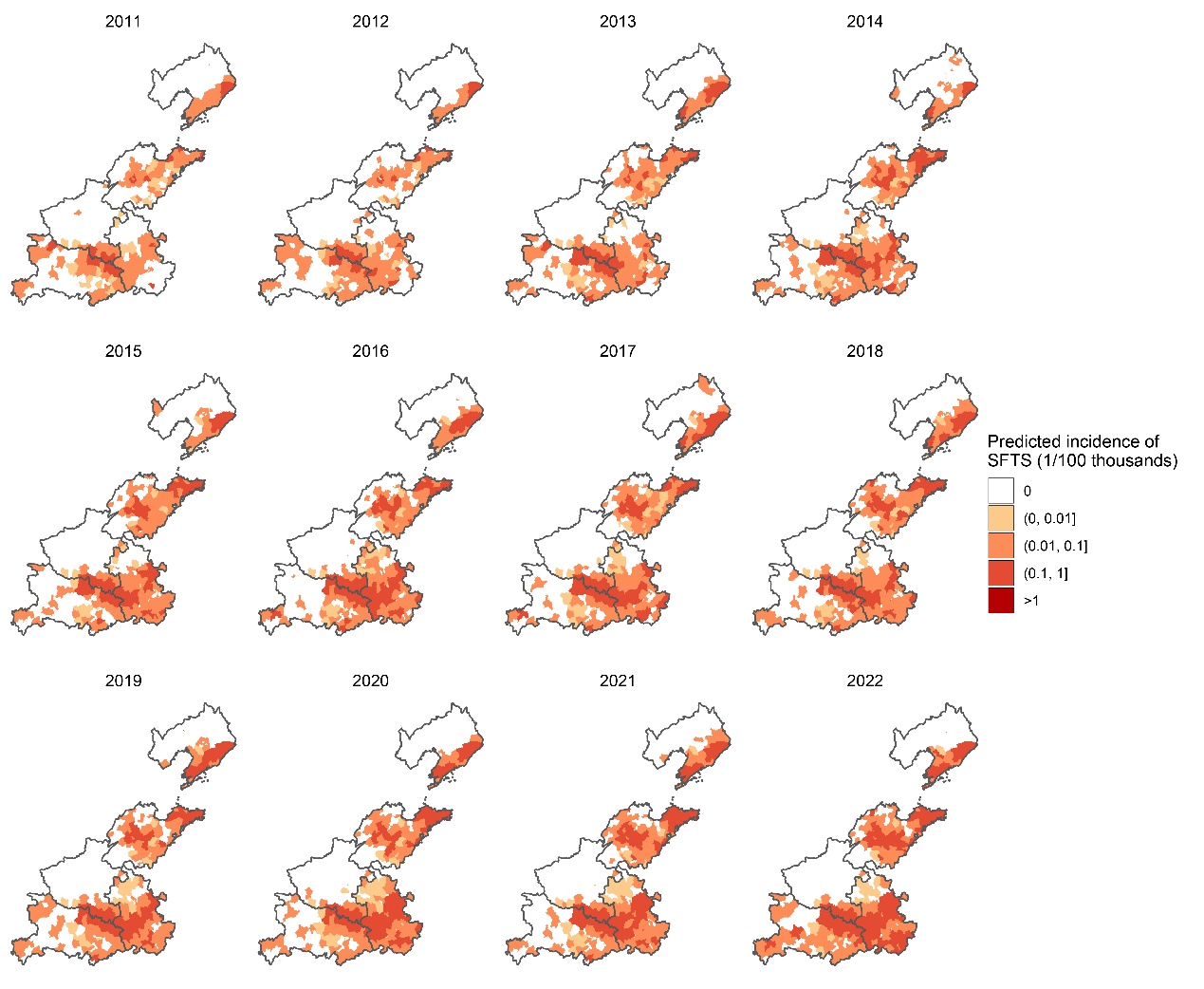


**Figure S9. Posterior predictive mean SFTS incidence rate from 2011-2022.**

Posterior predictive mean SFTS incidence rate per 100,000 people per year from 2011 to 2022 for the 604 counties in the five high-incidence provinces simulated from the Baseline-SPEI-TEMP-PREC model (refitted 12 × 12 times, leaving out one month per year at a time).


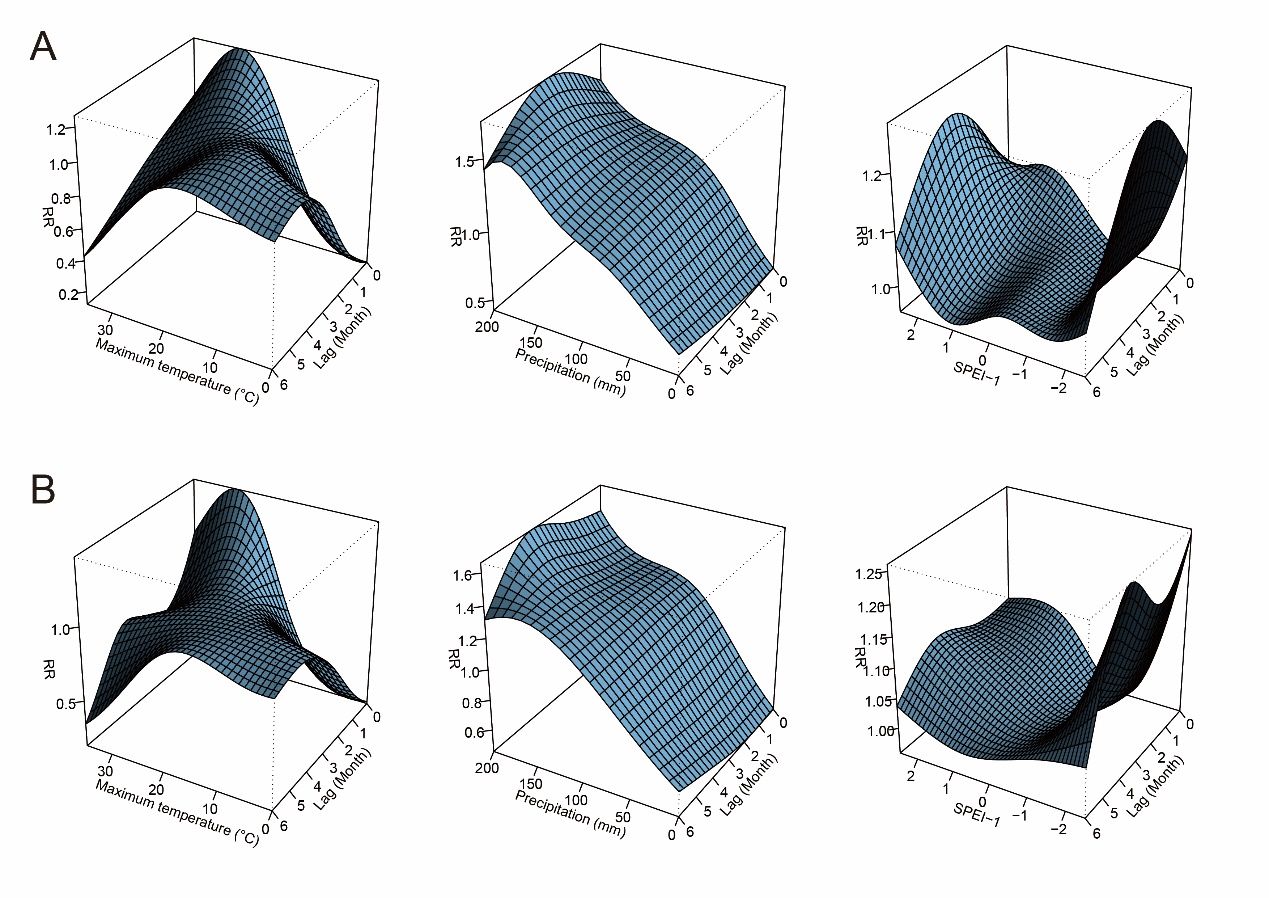


**Figure S10. Sensitivity results of relative risk of meteorological factors on the risk of SFTS.**

(A) The number of knots in the exposure dimension was changed to three. (B) The number of knots in the lag dimension was adjusted to two equally spaced internal knots.


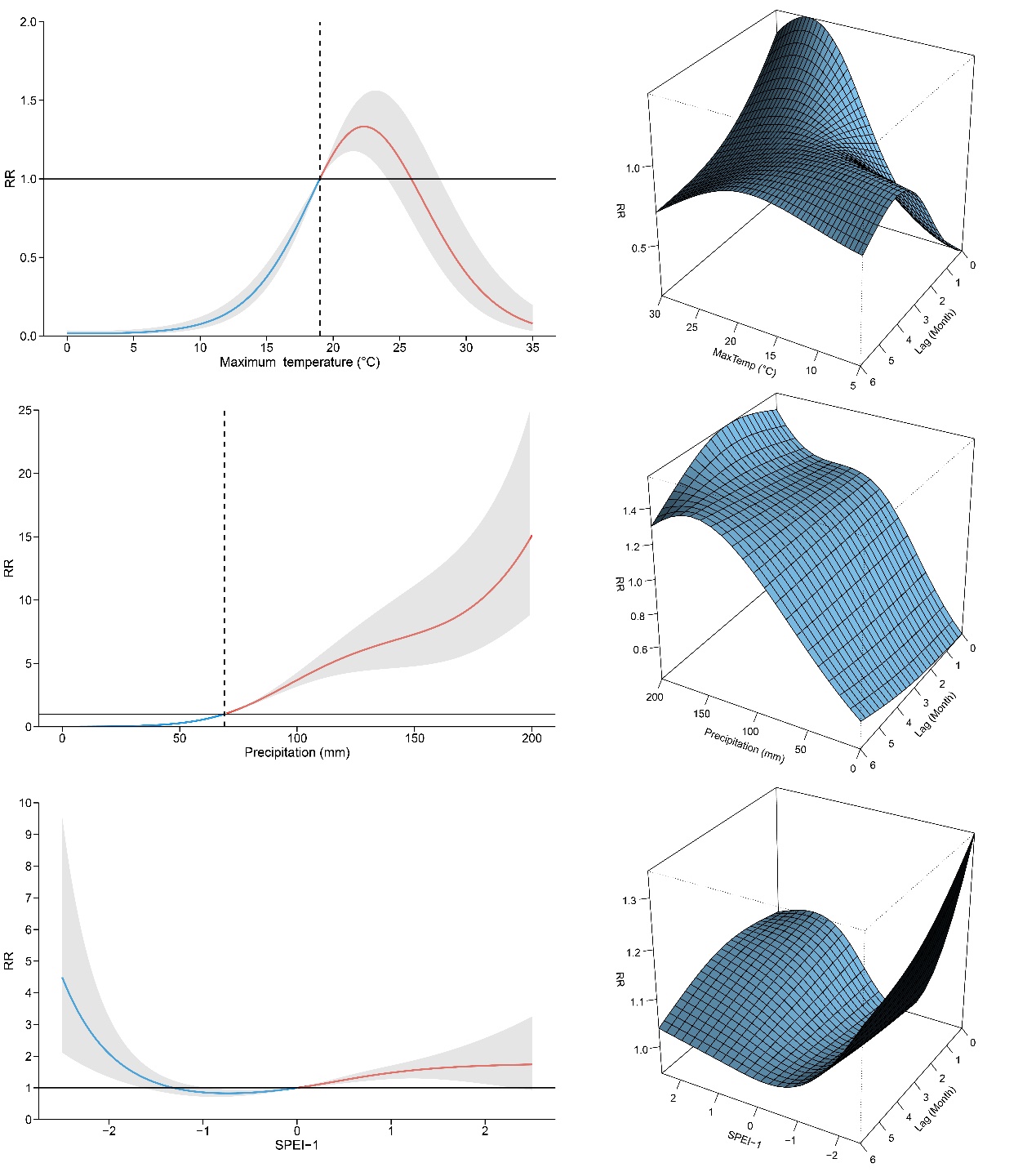


**Figure S11. Sensitivity analysis of underreporting in SFTS notifications.**


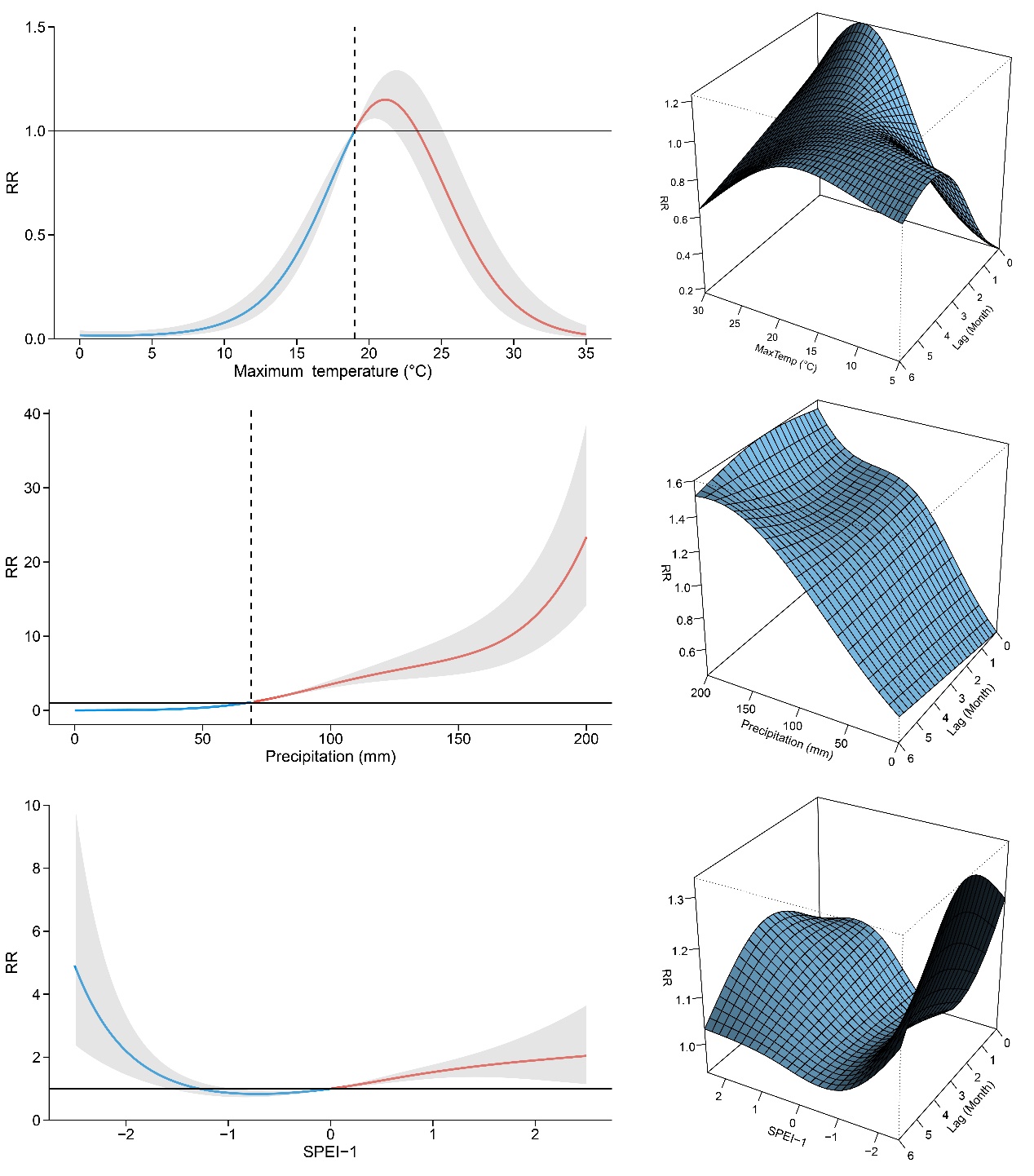


**Figure S12. Sensitivity analysis excluding the COVID-19 pandemic years.**


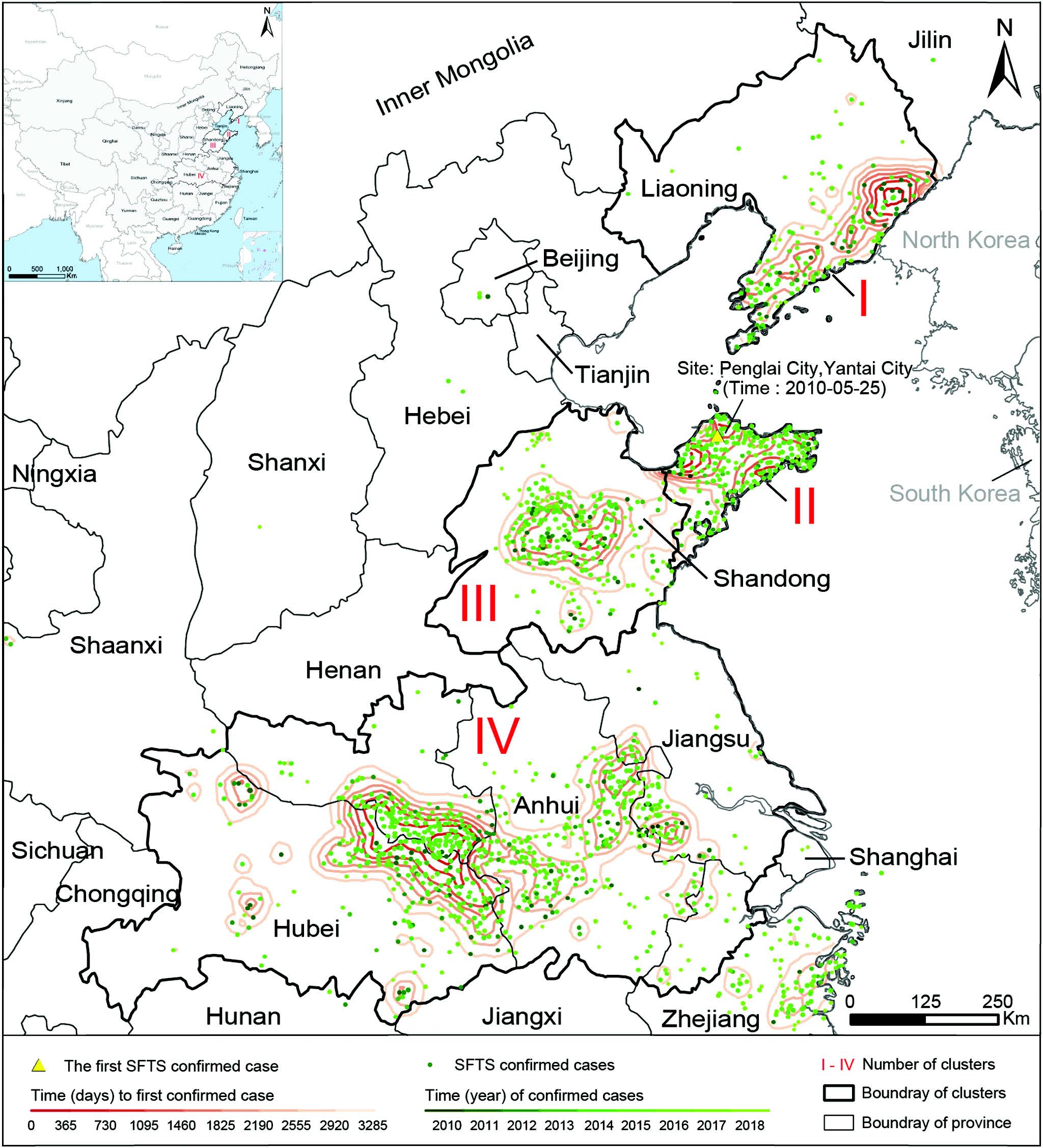


**Figure S13. Spatial distribution of four geographic clusters of SFTS cases in China.^1^**

1. D Miao, MJ Liu, YX Wang, et al. Epidemiology and Ecology of Severe Fever With Thrombocytopenia Syndrome in China, 2010‒2018. Clinical infectious diseases : an official publication of the Infectious Diseases Society of America. 2021;73(11):e3851-e8.


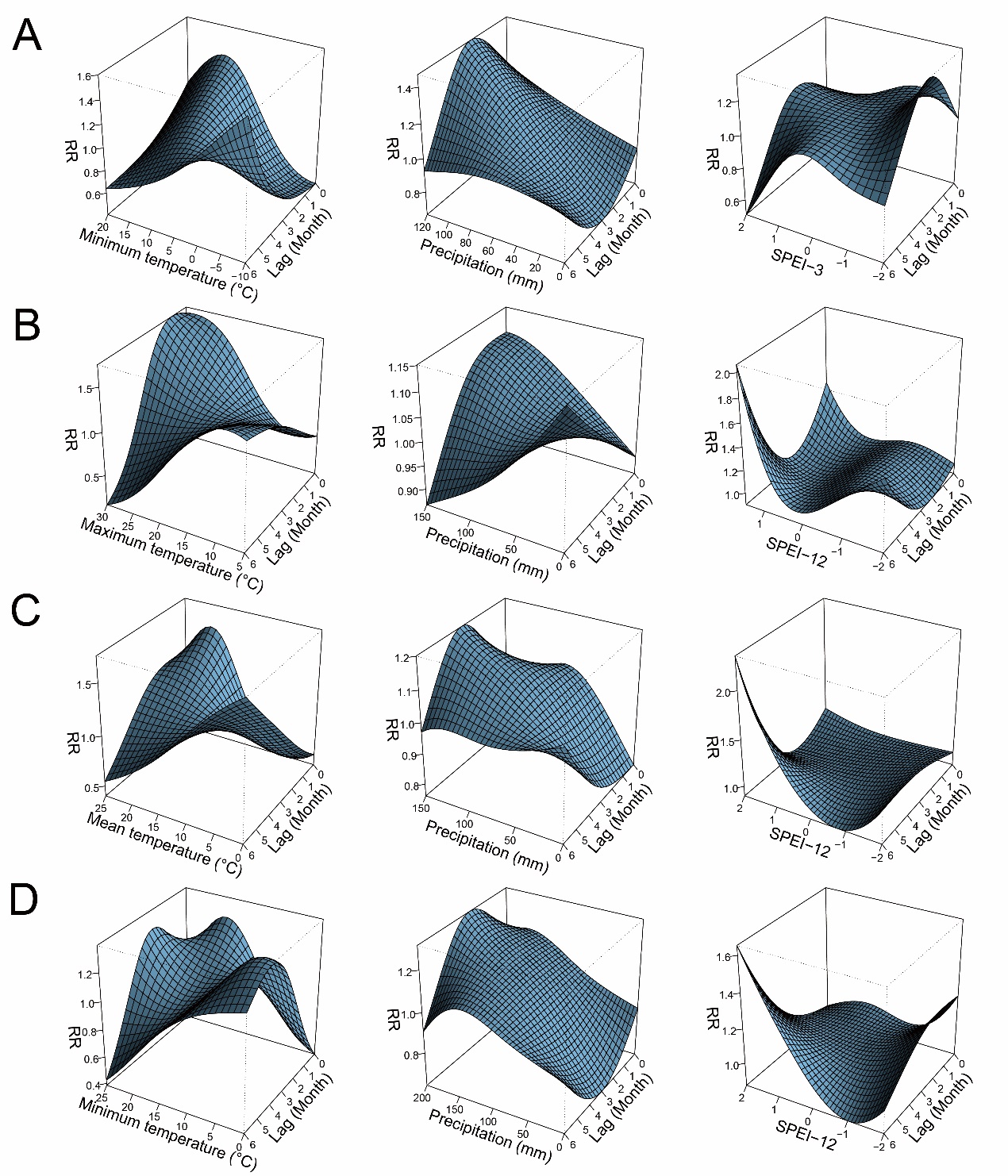


**Figure S14. 3D map of temperature, precipitation, and SPEI on the risk of SFTS in four geographical clusters.**

(A) Cluster I. (B) Cluster II. (C) Cluster III. (D) Cluster IV. SFTS, severe fever with thrombocytopenia syndrome; RR, relative risk; SPEI, Standardized Precipitation Evapotranspiration Index.


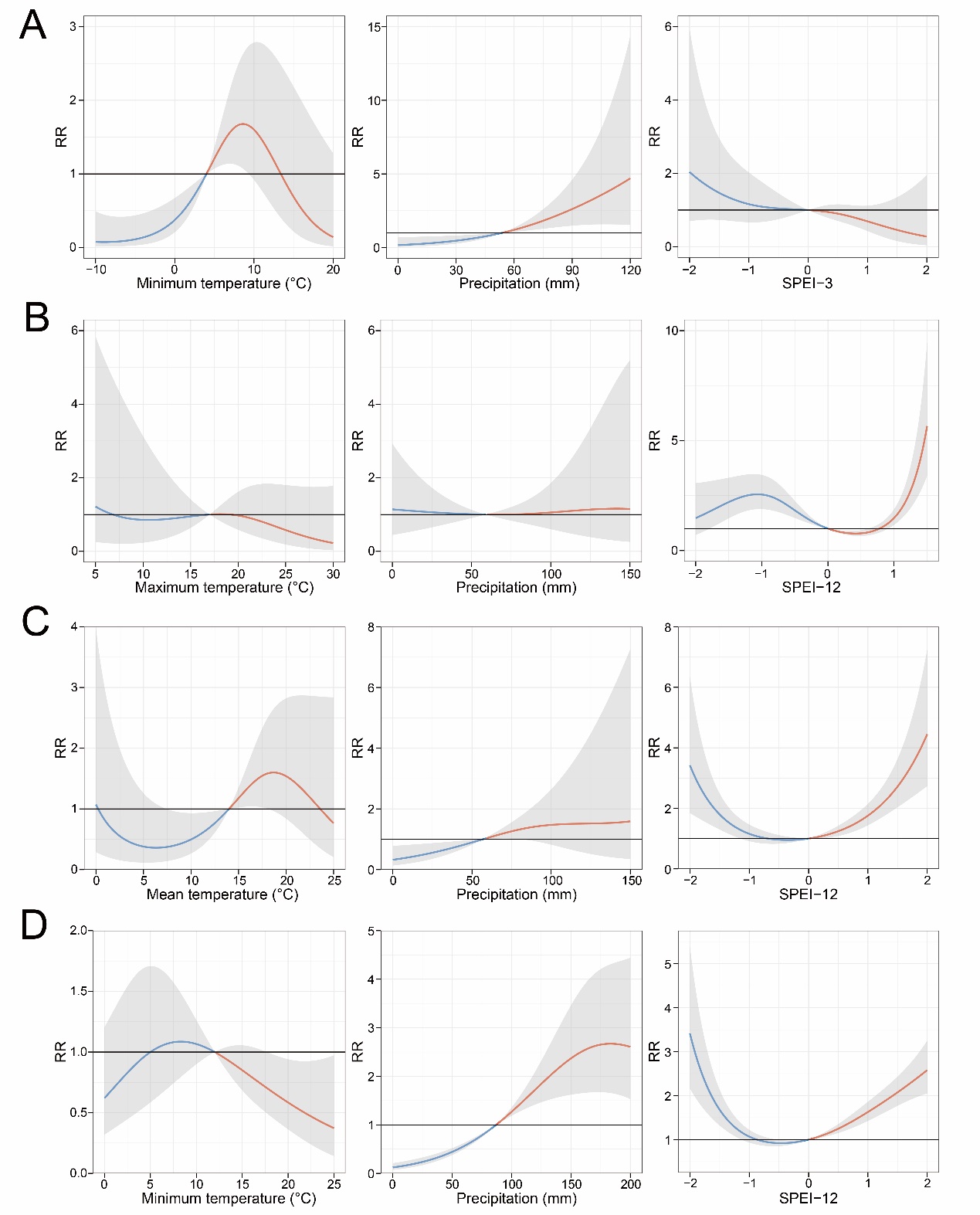


**Figure S15. Cumulative exposure-response effect of temperature, precipitation, and SPEI on the risk of SFTS in four geographical clusters.**

(A) Cluster I. (B) Cluster II. (C) Cluster III. (D) Cluster IV. The gray shading represents the 95% confidence interval. SFTS, severe fever with thrombocytopenia syndrome; RR, relative risk; SPEI, Standardized Precipitation Evapotranspiration Index.


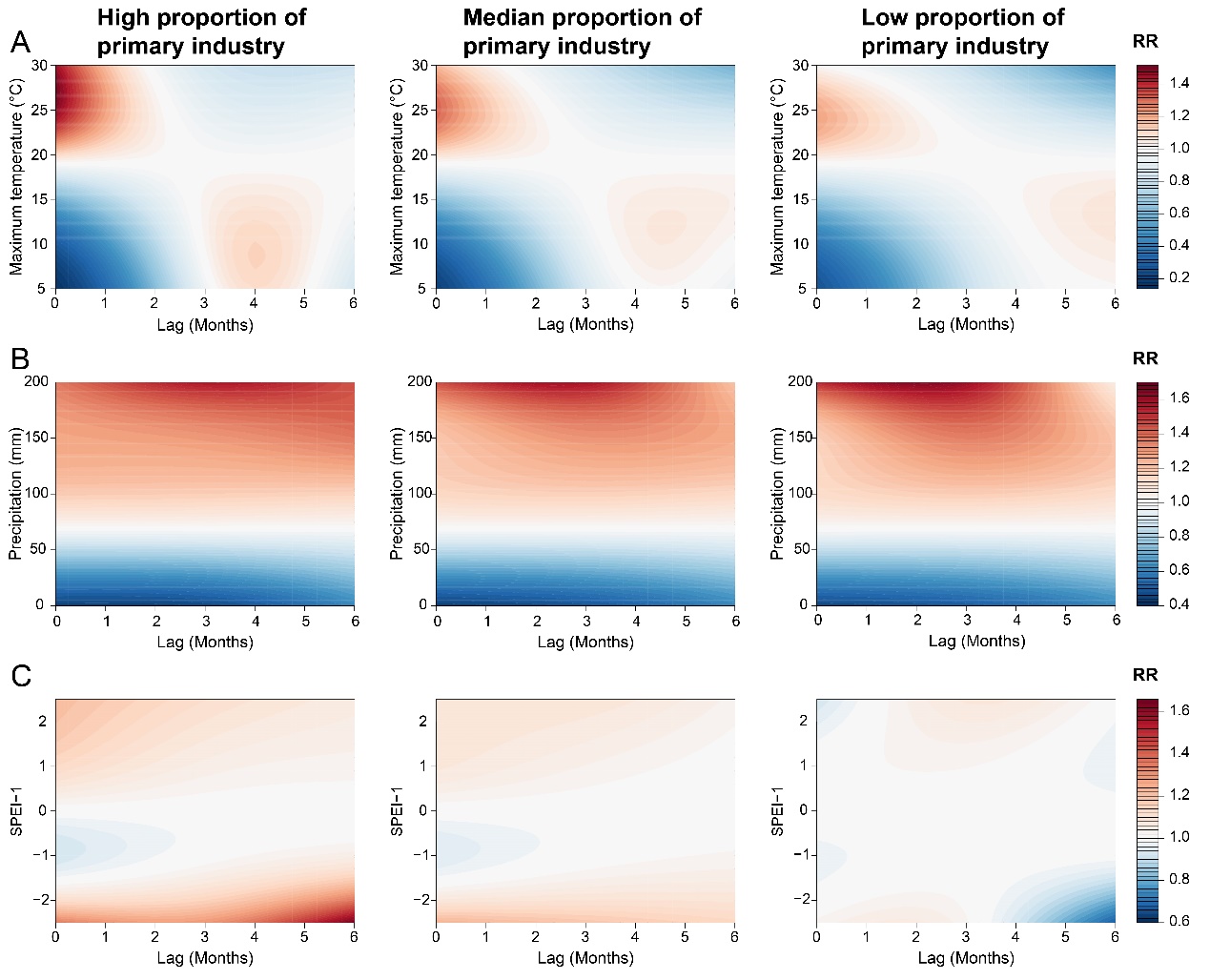


**Figure S16. Modification effects of proportion of value-added of primary industry on the association between SFTS incidence and meteorological factors.**

(A) Maximum temperature, (B) Precipitation, (C) SPEI-1.

Scenarios with a high proportion of value-added of primary industry, medium proportion of value-added of primary industry and low proportion of value-added of primary industry were demonstrated. The proportion of value-added of primary industry in the interaction term was centered on its 25th, 50th, and 75th percentile of the 604 counties’ value range. RR, relative risk; SPEI, Standardized Precipitation Evapotranspiration Index.


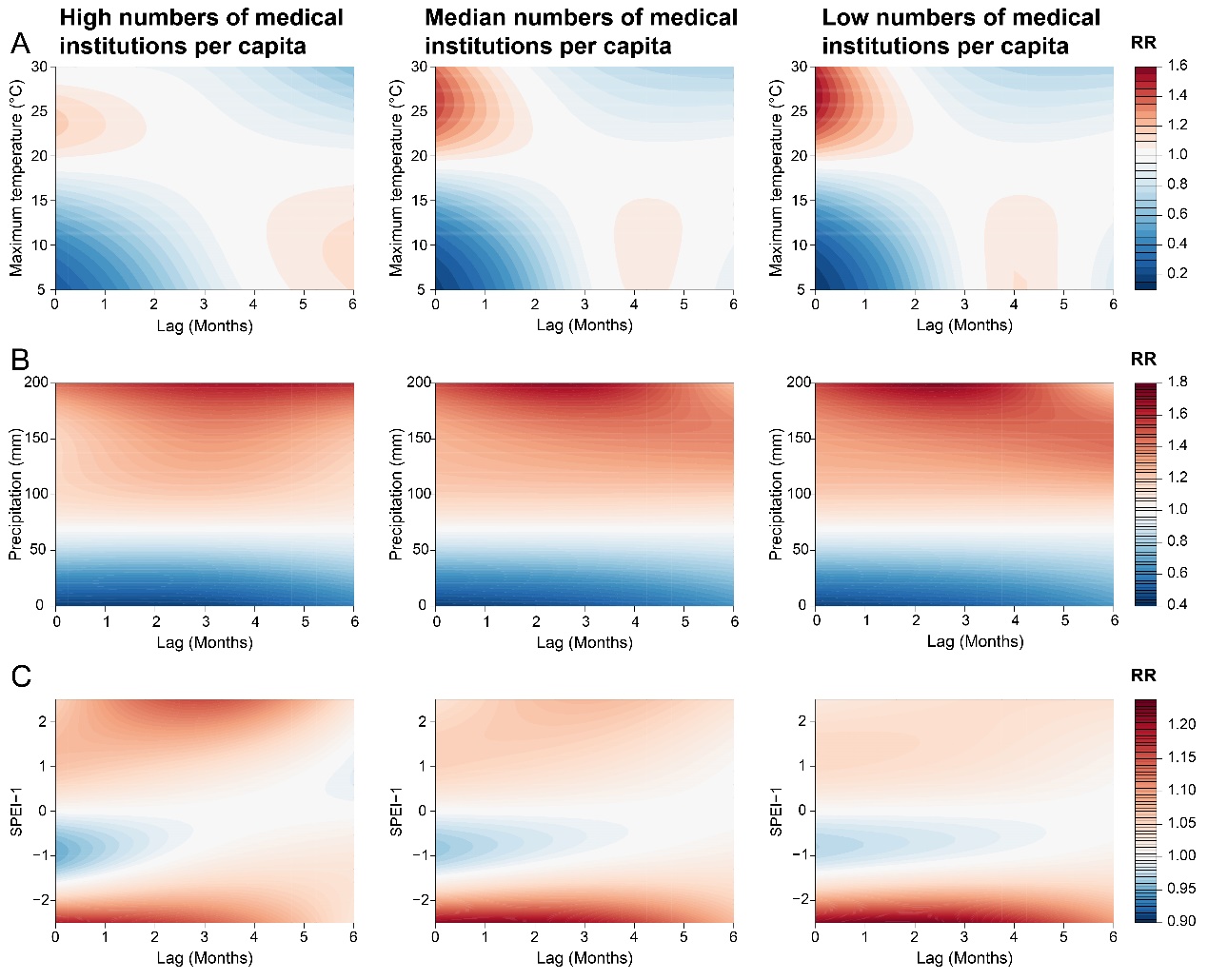


**Figure S17. Modification effects of numbers of medical institutions per capita on the association between SFTS incidence and meteorological factors.**

(A) Maximum temperature, (B) Precipitation, (C) SPEI-1.

Scenarios with a high proportion of numbers of medical institutions per capita, medium numbers of medical institutions per capita, and low numbers of medical institutions per capita were demonstrated. The numbers of medical institutions per capita in the interaction term was centered on its 25th, 50th, and 75th percentile of the 604 counties’ value range. RR, relative risk; SPEI, Standardized Precipitation Evapotranspiration Index.


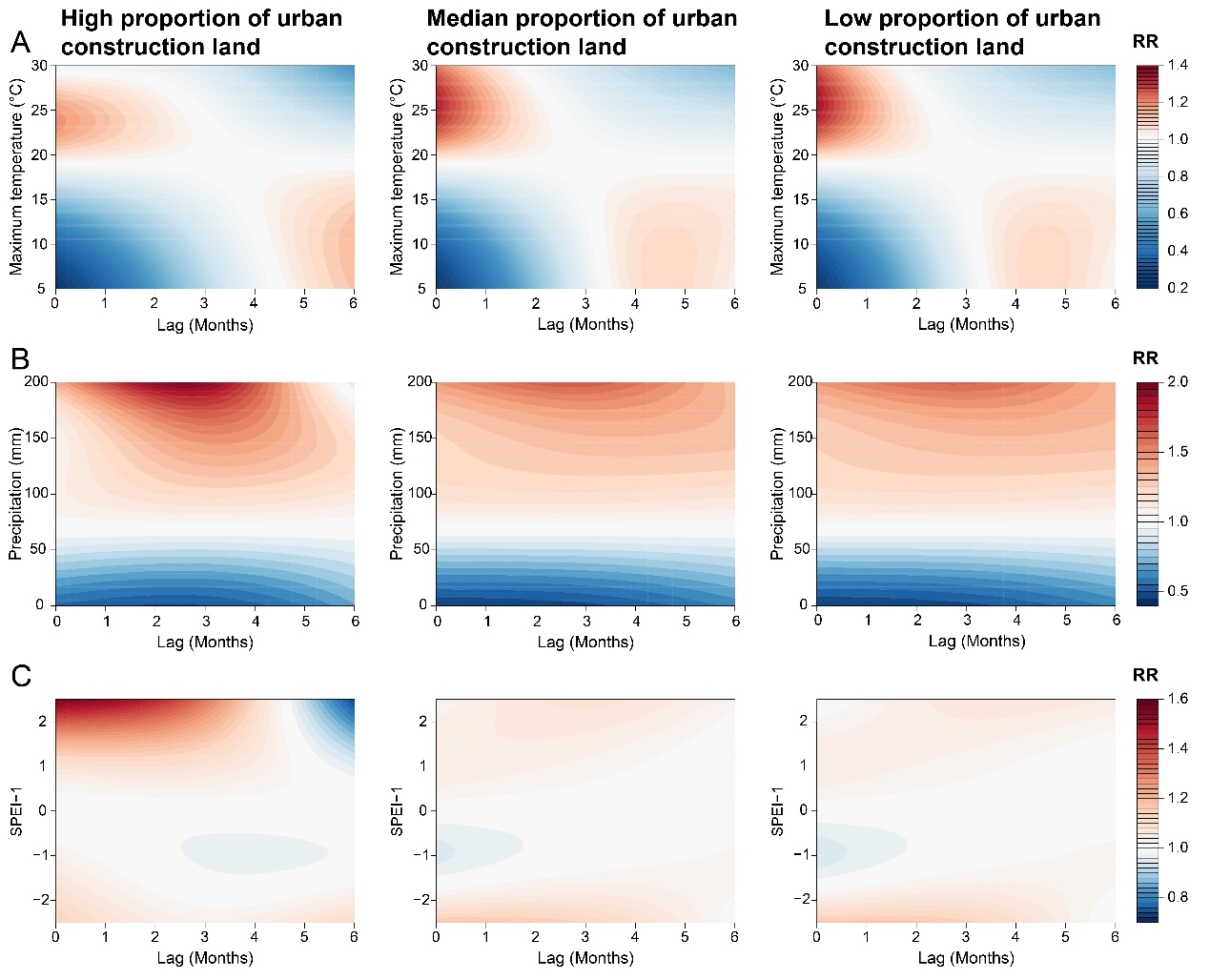


**Figure S18. Modification effects of proportion of urban construction land on the association between SFTS incidence and meteorological factors.**

(A) Maximum temperature, (B) Precipitation, (C) SPEI-1.

Scenarios with a high proportion of urban construction land, medium proportion of urban construction land, and low proportion of urban construction land were demonstrated. The proportion of urban construction land in the interaction term was centered on its 25th, 50th, and 75th percentile of the 604 counties’ value range. RR, relative risk; SPEI, Standardized Precipitation Evapotranspiration Index.


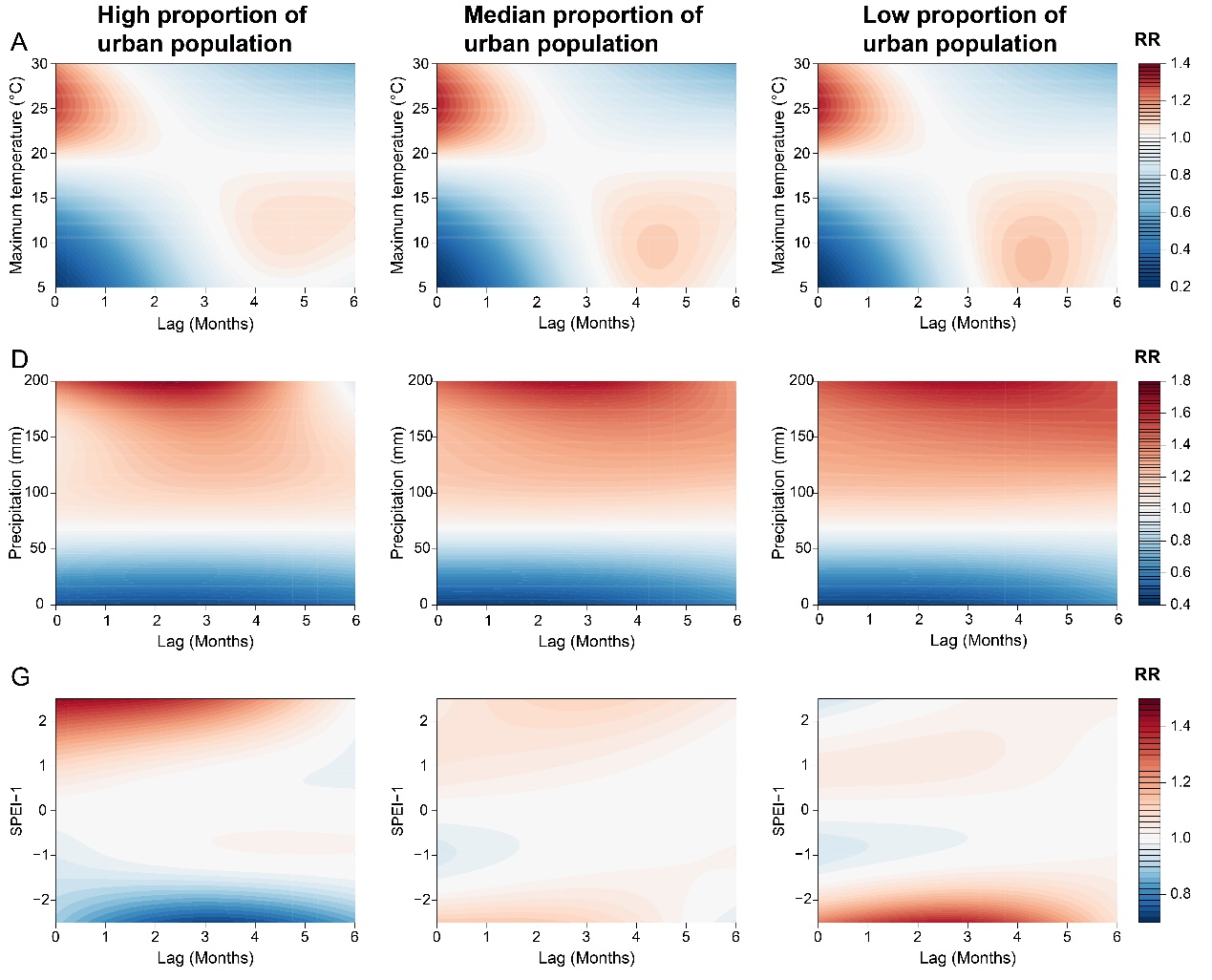


**Figure S19. Modification effects of proportion of urban population on the association between SFTS incidence and meteorological factors.**

(A) Maximum temperature, (B) Precipitation, (C) SPEI-1.

Scenarios with a high proportion of urban population, medium proportion of urban population, and low proportion of urban population were demonstrated. The proportion of urban population in the interaction term was centered on its 25th, 50th, and 75th percentile of the 604 counties’ value range. RR, relative risk; SPEI, Standardized Precipitation Evapotranspiration Index.
